# CD82 hypomethylation is essential for tuberculosis pathogenesis via regulation of RUNX1-Rab5/22

**DOI:** 10.1038/s12276-018-0091-4

**Published:** 2018-05-14

**Authors:** Hyun-Jung Koh, Ye-Ram Kim, Jae-Sung Kim, Jin-Seung Yun, Sojin Kim, Sun Young Kim, Kiseok Jang, Chul-Su Yang

**Affiliations:** 1 Department of Molecular and Life Science, Hanyang University, Ansan, 15588 South Korea; 2 Department of Bionano Technology, Hanyang University, Seoul, 04673 South Korea; 3 Department of Pathology, Hanyang University College of Medicine, Seoul, 04763 South Korea

## Abstract

The tumor suppressor gene CD82/KAI1 is a member of the tetraspanin superfamily and organizes various membrane-based processes. *Mycobacterium tuberculosis* (MTB) persists in host macrophages by interfering with phagolysosome biogenesis and inflammatory responses, but the role of CD82 in controlling the intracellular survival of pathogenic mycobacteria within macrophages remains poorly understood. In this study, we demonstrated that the virulent MTB strain H37Rv (MTB Rv) induced CD82 promoter hypomethylation, resulting in CD82 expression. Targeting of the runt-related transcription factor 1 (RUNX1) by CD82 is essential for phagosome arrest via interacting with Rab5/22. This arrest is required for the intracellular growth of MTB in vitro and in vivo, but not for that of MTB H37Ra (MTB Ra) in macrophages. In addition, knockdown or knockout of CD82 or RUNX1 increased antibacterial host defense via phagolysosome biogenesis, inflammatory cytokine production, and subsequent antimicrobial activity both in vitro and in vivo. Notably, the levels of CD82 and RUNX1 in granulomas were elevated in tuberculosis (TB) patients, indicating that CD82 and RUNX1 have clinical significance in human TB. Our findings identify a previously unrecognized role of CD82 hypomethylation in the regulation of phagosome maturation, enhanced intracellular survival, and the innate host immune response to MTB. Thus, the CD82–RUNX1–Rab5/22 axis may be a previously unrecognized virulence mechanism of MTB pathogenesis.

## Tuberculosis: Evading host defences

The tuberculosis-causing bacterium *Mycobacterium tuberculosis* regulates a tumor suppressor gene in order to survive and grow in host immune cells. Chul-Su Yang and colleagues at Hanyang University, South Korea, have found that the bacterium can stimulate the expression of *CD82* in macrophages by removing methyl groups from its DNA sequence. *CD82*’s hypomethylated region interacts with and activates proteins that interfere with the cell’s ability to mount an inflammatory response and degrade bacteria in specialized intracellular vesicles called lysosomes. The increased survival rate of CD82-deficient mice following infection with tuberculosis and the elevated levels of CD82 protein found in the inflammatory lesions of patients with tuberculosis further support a previously unrecognized role for this protein in *M. tuberculosis* infection. Targeting *CD82*-mediated signaling could be a promising approach for designing new therapeutics.

## Introduction

CD82/KAI1, a member of the tetraspanin family, is a cancer metastasis suppressor that has been implicated in diverse biological processes, including fusion, adhesion, migration, apoptosis, and cell morphology alteration^1,2^. The downregulation of CD82 expression is associated with advanced stages of several human cancers and correlates with the acquisition of metastatic potential^1–3^. Recent studies have suggested that the anti-metastatic ability of CD82 is attributable to: (i) its interaction with cellular targets, such as integrin, the Duffy antigen receptor for chemokines on endothelial cells, epidermal growth factor receptor, and signaling molecules; and (ii) the complex mechanisms underlying CD82 loss of function, including altered transcriptional regulation, splice variant production, and post-translational protein modifications^1–4^. Taken together, these functions indicate a central role for CD82 in controlling metastasis as a “molecular facilitator.” CD82 is also associated with components of the major histocompatibility complex (MHC) class II antigen presentation pathway, including class II MHC molecules and the peptide-loading machinery, and is specifically recruited to pathogen-containing phagosomes in macrophages prior to fusion with lysosomes^5,6^, suggesting roles for CD82 in antigen presentation and intracellular trafficking in macrophages. The regulation of CD82 gene expression is becoming an increasingly important issue. Altered regulatory mechanisms have been strongly linked to mutations in, or allelic losses of, the CD82 gene in prostate tumors^1,4^. The transcriptional silencing of genes is caused by epigenetic mechanisms, including DNA methylation and histone modification, as well as by regulation via non-coding RNAs, which play pivotal roles in disease development^7–9^. However, neither the involvement of such an epigenetic regulation of CD82 expression in tuberculosis (TB) pathogenesis nor the mechanisms by which CD82 is involved in controlling the intracellular survival of pathogenic mycobacteria in macrophages are well understood.

*Mycobacterium tuberculosis* (MTB), the causative pathogen of the infectious and contagious disease TB, is a well-known intracellular parasite that can evade host immunity and survive for long periods of time within macrophages^10,11^. The survival strategies of MTB inside the macrophage include: (i) the inhibition of vesicular trafficking and membrane fission/fusion events responsible for phagocytosis and phagolysosome maturation, (ii) the inhibition of apoptosis and autophagy, (iii) the inhibition of the macrophage response to proinflammatory cytokines including interferon-γ (IFN-γ) and innate signaling pathways, (iv) the inhibition of MHC class II expression on macrophages to prevent antigen presentation to CD4+ T cells, (v) the disruption of cytoskeletal organization, (vi) the inhibition of reactive oxygen/nitrogen intermediate generation, and (vii) the inhibition of host antimicrobial peptides^12–16^. Thus, although MTB has evolved several mechanisms to facilitate survival within macrophages, the regulation of these mechanisms remains poorly understood.

Since the complete genome of MTB was sequenced in 1998^17^, several comparative studies have been performed investigating differentially expressed genes between the virulent MTB Rv and the avirulent MTB Ra aimed at identifying mycobacterial virulence factors^18–21^. Although a recent study using DNA microarrays and messenger RNA (mRNA) differential-display assays to explore macrophage activation and the cytokine/chemokine response to infection with different MTB strains has described the ability of cells to control the growth of intracellular bacilli^21–23^, little is known regarding the differential expression of host genes under pathophysiologic conditions induced in response to different strains of mycobacteria. Thus, we used RNA-sequencing (RNA-seq) analysis to identify possible factors in host macrophages determining the differential pathological responses to MTB Rv and MTB Ra in an in vivo model of mycobacterial infection.

In this study, we investigated the intracellular regulatory network of virulent MTB-induced aberrant epigenetic regulation of CD82, which contributes to the pathogenesis of TB via runt-related transcription factor 1 (RUNX1)–Rab5/22. We found that promoter demethylation of CD82 was essential for interaction with RUNX1 in the nucleus and with Rab5/22 in the phagosome, both of which contribute to MTB virulence in vitro and in vivo. Notably, levels of CD82 and RUNX1 were significantly elevated in granulomas of TB patients, indicating their clinical significance in human TB. Thus, the CD82–RUNX1–Rab5/22 axis may be an important virulence mechanism of MTB pathogenesis.

## Materials and methods

### Ethics statement

All animal experimental procedures were reviewed and approved by the Institutional Animal Care and Use Committee of Hanyang University (protocol 2014-0207) and Bioleaders Corporation (Daejeon, Korea, protocol BLS-ABSL3-14-11). All animal experiments were performed in accordance with the Korean Food and Drug Administration guidelines.

### Bacterial strains and preparation of *Mycobacterium* spp

MTB H37Rv (ATCC 27294), MTB H37Ra (ATCC 25177), *M. bovis* BCG (ATCC 19274) and *M. smegmatis* (ATCC 19420) were purchased from American Type Culture Collection (ATCC, Manassas, VA, USA). All mid-log mycobacteria (OD_600_ = 0.5–0.6) used in this study were prepared as described previously^24^.

### Mice and cell culture

Wild-type C57BL/6 mice were purchased from Orient Bio (Gyeonggi-do, Korea). RUNX1-floxed mice (B6.129P2-*Runx1*^*tm1Tani*^/J, 008772) and PLD1^−/−^ (B6.129P2-*Lyz2*^*tm1(cre)Ifo*^/J, 004781) mice were obtained from the Jackson Laboratory. Cre-mediated recombination was confirmed by PCR using genomic DNA from isolated peritoneal macrophages and primers flanking the floxed region, as described previously^25^; the absence of RUNX1 expression in these macrophages was confirmed by immunoblot analysis using a RUNX1-specific antibody (Santa Cruz, C-19, Dallas, TX, USA). CD82 gene (Transcript: ENSMUSG00000027215) knockout (KO) mice were generated using CRISPR/Cas9-mediated genome editing. CRISPR/Cas9 single-guide RNAs (sgRNAs) and Cas9 protein were designed and synthesized by ToolGen, Inc. (Seoul, Korea), and sgRNA and Cas9 mRNA were co-injected into C57BL/6 mouse embryos to create KO mice. The F0 founders had a deletion of exons 5 and 6 resulting from the gRNA1/Cas9 procedure. PCR genotyping, sequencing, and in vitro T7 endonuclease I digestion assays were performed to verify the frame shift changes for CD82 KO mice. PCR primers for mouse tail genotyping are shown in Table 1. All animals were maintained in a specific pathogen-free environment. HEK293T cells (ATCC-11268) and J774A.1 cells (ATCC TIB-67) were maintained in Dulbecco's modified Eagle's medium (DMEM; Invitrogen, Waltham, MA, USA) containing 10% fetal bovine serum (FBS; Invitrogen), sodium pyruvate, nonessential amino acids, penicillin G (100 IU/ml), and streptomycin (100 μg/ml). Human monocytic THP-1 (ATCC TIB-202) cells were grown in RPMI-1640/glutamax supplemented with 10% FBS. Primary bone marrow-derived macrophages (BMDMs) were isolated from C57BL/6 mice and cultured in DMEM for 3–5 days in the presence of 25 ng/ml recombinant macrophage colony-stimulating factor (R&D Systems, 416-ML, Minneapolis, MN, USA), as described previously^26^. Transient transfections were performed using Lipofectamine 2000 (Invitrogen), or calcium phosphate (Clontech, Mountain View, CA, USA), according to the manufacturer’s instructions. J774A.1 stable cell lines were generated using a standard selection protocol with 400–800 μg/ml of G418.

**Table 1:** Primers used in this study

***Gene***	***Sense primer***	***Antisense primer***	***Size (bp)***	***Tm (°C)***
mCD82 genotyping	CAC CCT CCT GCA CTC AAT CT	CCA CCT GTG ACA ACC AAG TG	612	60
mCD82 methylation 1	GTT ATT GTT TTT CGT GTG AGA TTT C	ACC TTC CAT ATT ATT AAA ACA CCG A	159	65.7
mCD82 Un-methylation 1	TTA TTG TTT TTT GTG TGA GAT TTT GA	CCT TCC ATA TTA TTA AAA CAC CAA A	157	65.2
mCD82 methylation 2	TCG TAG ATT TAT TTT AGG GGT GTT C	AAT CTA CCT ATA TTT CCT TCC CGA C	182	66.5
mCD82 Un-methylation 2	TGT AGA TTT ATT TTA GGG GTG TTT GT	AAT CTA CCT ATA TTT CCT TCC CAA C	181	66.1
mdnmt1	GGG TCT CGT TCA GAG CTG	GCA GGA ATT CAT GCA GTA AG	201	60
mdnmt2	CCG CCT CTT CTT TGA GTT CTA C	AGA TGT CCC TCT TGT CAC TAA CG	125	55
mdnmt3	ATG GAG ATC AGG AGG GTA TGG A	GTC GCT TGG AGG TGG CTT TC	177	56
mtet1	GCA CCC CAA CC TAA TCA TC	ACC TTC ATT AGC TGC CTG GT	172	58
mtet2	ACA GAA GCA AGA ACA GCA GC	AGC TTG CAG GTG GAT TCT CT	176	59
mtet3	CCC CTT CCC ACT TCA CG AT	CAC AGC TTG TCT TGG AAC CC	229	59
mβ-actin	AAG TGT GAC GTT GAC ATC CG	GAT CCA CAT CTG CTG GAA GG	222	58
mCD82 ChIP-PCR	GCC ATT GTT TCC CGT GTG AGA CCT CG	TGC CTT CCA TGT TGT TGA GGC ACC GG	160	58

### *M. tuberculosis* infection in vitro and in vivo

For in vitro experiments, cells were infected with MTB (multiplicity of infection (MOI) = 1, 5, or 10) for 2–4 h. Then, cells were washed with DMEM, and BMDMs were pulsed with 200 μg/ml amikacin (Sigma-Aldrich) to kill extracellular mycobacteria^13^. After 1 h, BMDMs were washed with DMEM to remove all extracellular bacteria and cultivated in 1% FBS–DMEM containing 20 μg/ml amikacin and incubated at 37 °C in 5% CO_2_ for the indicated time points. For in vivo experiments, KO and C57BL/6 mice were intravenously injected with MTB (1 × 10^8^ colony-forming units (CFUs)/mouse) or intranasally injected with MTB (1 × 10^3^ CFUs/mouse)^26,27^. After 3 weeks of infection, mice were killed, and the lungs and sera were harvested. All mice were maintained in biosafety level 3 laboratory facilities.

### Reagents and plasmids

5-Aza-2′-deoxycytidine (A3656) and dimethyl sulfoxide (DMSO) were purchased from Sigma-Aldrich (Irvine KA, MO, USA). pEYFP-CD82 was obtained from Addgene (Cambridge, MA, USA). pEGFP-Rab5 wld type (WT), Q67L, and S34N were a generous gift from Dr. Michel J. Tremblay (Laval University, Canada). pEGFP-Rab22 WT, Q64L, and S19L were a generous gift from Dr. P.D. Stahl (Washington University School of Medicine, USA). Hemagglutinin (HA)- or glutathione *S*-transferase (GST)-tagged CD82 and truncated mutant genes were described previously^26^. Flag-tagged Rabptin-5 or Rabenosyn-5 genes were each cloned into the *Xba*I and *Bam*HI sites in the pcDNA3.0 vector. All constructs were sequenced using an ABI PRISM 3730XL automatic DNA sequencer to verify 100% correspondence with the original sequence.

### Immunoblot analysis and immunoprecipitation

THP-1, 293T, J774A.1, and BMDM cells were treated and processed for analysis by western blotting, co-immunoprecipitation, and GST pulldown as previously described^26,28^.

### Transcriptome sequencing (RNA-Seq)

Total RNA was extracted using RNAiso Plus (Takara Bio, Inc., Kusatsu, Shiga, Japan). Extracted RNA was purified using the RNeasy Mini Kit accompanied by DNase I (Qiagen, Germantown, MD, Germany) treatment. RNA-seq libraries were generated using the TruSeq RNA sample Preparation Kit (Illumina, San Diego, CA, USA), and complementary DNA libraries were sequenced on a HiSeq 2000 (Illumina) to obtain approximately 100 million paired-end reads (2 × 101 bp).

### Differentially expressed gene analysis using RNA-seq data

FASTQ files from RNA-seq experiments were clipped and trimmed of adapters, and low-quality reads were removed using Trimmomatic^29^. Quality-controlled FASTQ files were aligned to the UCSC hg19 reference genome using STAR (version 2.5.1) aligner software^30^ with three mismatches. To measure differential gene expression, DESeq2^31^ with the default parameters was used. A subset of condition-specific expression was defined as showing a 1.0 log_2_-fold difference and *P*-adj < 0.01 in expression between MTB Rv and MTB Ra. RNA-seq experiments were normalized and visualized using HOMER^32^ after preparing custom tracks for the UCSC Genome Browser (http://genome.ucsc.edu/).

### Histology

For immunohistochemistry analysis of tissue sections, murine lungs were fixed in 10% formalin and embedded in paraffin. Paraffin sections (4 μm) were cut and stained with hematoxylin and eosin (H&E) or CD82 and RUNX1 antibodies^33^.

### Statistical analysis

All data were analyzed using Student’s *t-*test with Bonferroni adjustment or analysis of variance (ANOVA) for multiple comparisons and are presented as the means ± SD. Grubbs’ test was used for evaluating outliers. Statistical analyses were conducted using the SPSS (Version 12.0) statistical software program (SPSS, Chicago, IL, USA). Differences were considered significant at *P* < 0.05. For survival, data were graphed and analyzed by the product limit method of Kaplan and Meier, using the log-rank (Mantel–Cox) test for comparisons using GraphPad Prism (version 5.0, La Jolla, CA, USA).

## Results

### CD82 contributes to TB pathogenesis

**Fig. 1:**
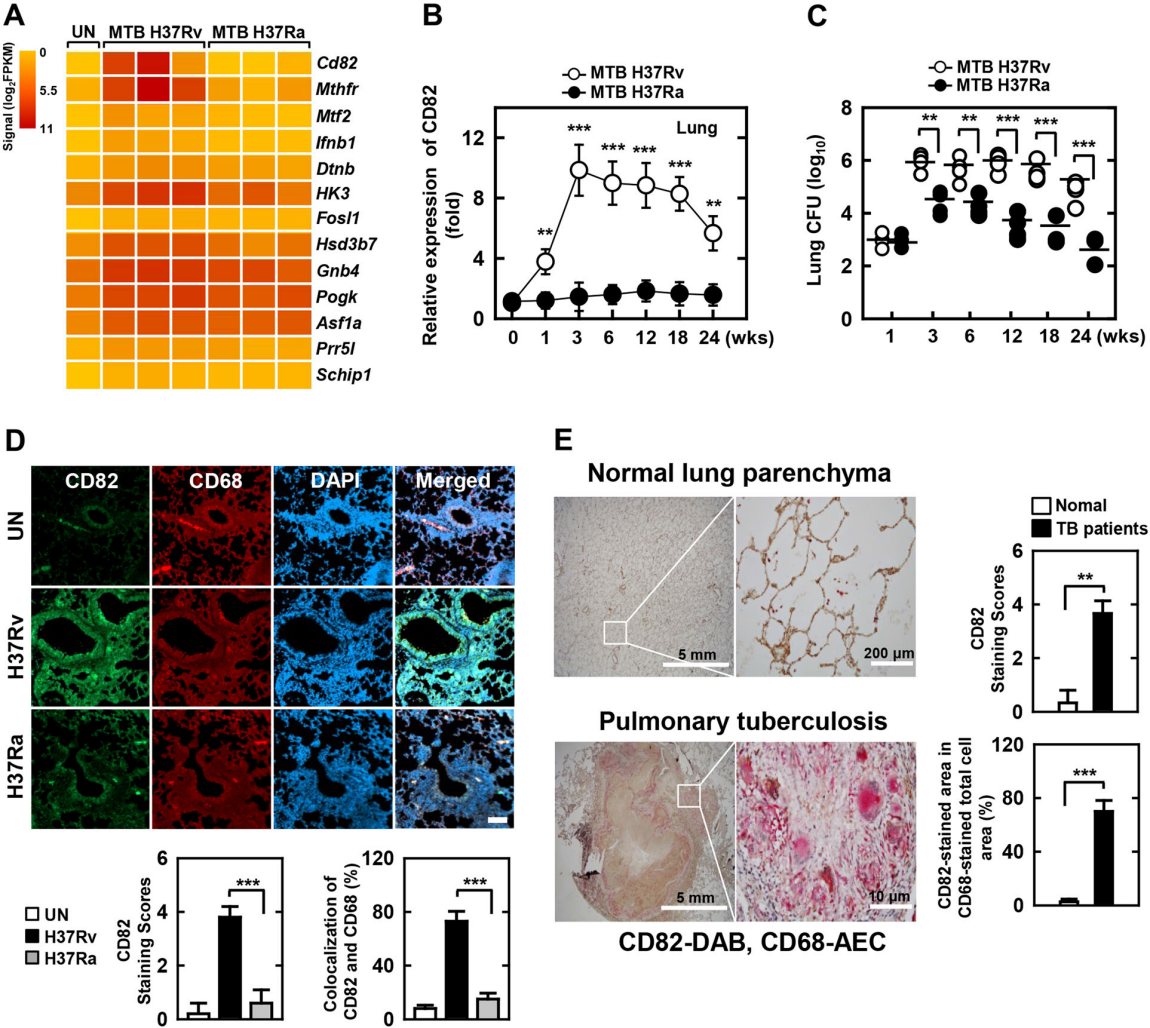
Identification of MTB Rv-specific CD82 in mice and TB patients. **a** Heat map of the top 13 upregulated genes in lungs from MTB Rv- or MTB Ra-infected mice. Each row shows the relative expression level of a single gene, and each column shows the expression level of a single sample. **b** Real-time qPCR analysis of CD82 expression or **c** bacterial loads in lungs from MTB Rv- or MTB Ra-intranasal infected mice for the indicated times (as in Fig. S1A). **d** Representative immunofluorescence images for expression of CD82 and CD68 (a macrophage marker) in lungs from MTB Rv- or MTB Ra-infected mice. Scale bar, 100 μM. The bottom panel shows the quantitative data of staining intensity of CD82 (left) and the colocalization index (%) between CD82 and CD68 (right). **e** Immunohistochemical analysis to examine CD82-DAB (3,3’-diaminobenzidine) and CD68-AEC (3-amino-9-ethylcarbazole) expression in healthy controls and patients with pulmonary TB. Representative images from five independent healthy controls and patients are shown. Insets, enlargement of outlined areas. Biological replicates (*n* = 3) for each condition were performed (**a**–**d**). Significant differences (**P *<* 0.01; ***P *<* 0.001) compared with MTB Ra (Student’s *t-*test with Bonferroni adjustment)

To identify the MTB virulence mechanisms involved in controlling intracellular survival of pathogenic mycobacteria in MTB-infected macrophages, we conducted differential gene expression profiling of murine lungs exposed to MTB Rv or MTB Ra using RNA-seq analysis. As previously reported^27,34^, we selected a 3-week time point for whole-genome transcriptional profiling in MTB-infected mice (Fig. S1A). Three independent samples (biological replicates) for each treatment were processed. We used a 1% false discovery rate, *P* < 0.001 and a fold change of >1.0 log_2_ for up- or downregulation as the criteria for defining differentially expressed genes. RNA-seq and quantitative real-time PCR analyses showed that CD82/KAI1 were the most significantly upregulated genes expressed in MTB Rv versus MTB Ra infection (Fig. 1a, b and S1B). Previous findings have demonstrated that MTB Rv CFUs significantly increased and MTB Ra growth was better controlled by macrophages^22,34^, and we confirmed these findings in a murine model of established TB that resembles human TB (Fig. 1c and S1C). The expression of CD82 in lung tissues was markedly increased in macrophages of MTB Rv-infected mice but not in MTB Ra-infected mice (Fig. 1d, and S1D). Furthermore, histological analysis revealed the presence of macrophage-associated acid-fast bacteria in granulomas (Fig. S1E).

We further examined pulmonary CD82 expression between healthy controls and patients with pulmonary TB (Fig. 1e). CD82 expression was weakly positive in the cytosol of epithelial cells of bronchioles, pneumocytes, and smooth muscle cells of blood vessels from normal lung parenchyma; however, pulmonary TB showed strong positivity in epithelioid cells and multinucleated giant cells in granulomas. These results show that virulent MTB-induced CD82 expression in macrophages is clinically significant in human TB.

### CD82 is essential for reduced inflammation and enhanced bacterial growth in virulent mycobacterial infection

**Fig. 2:**
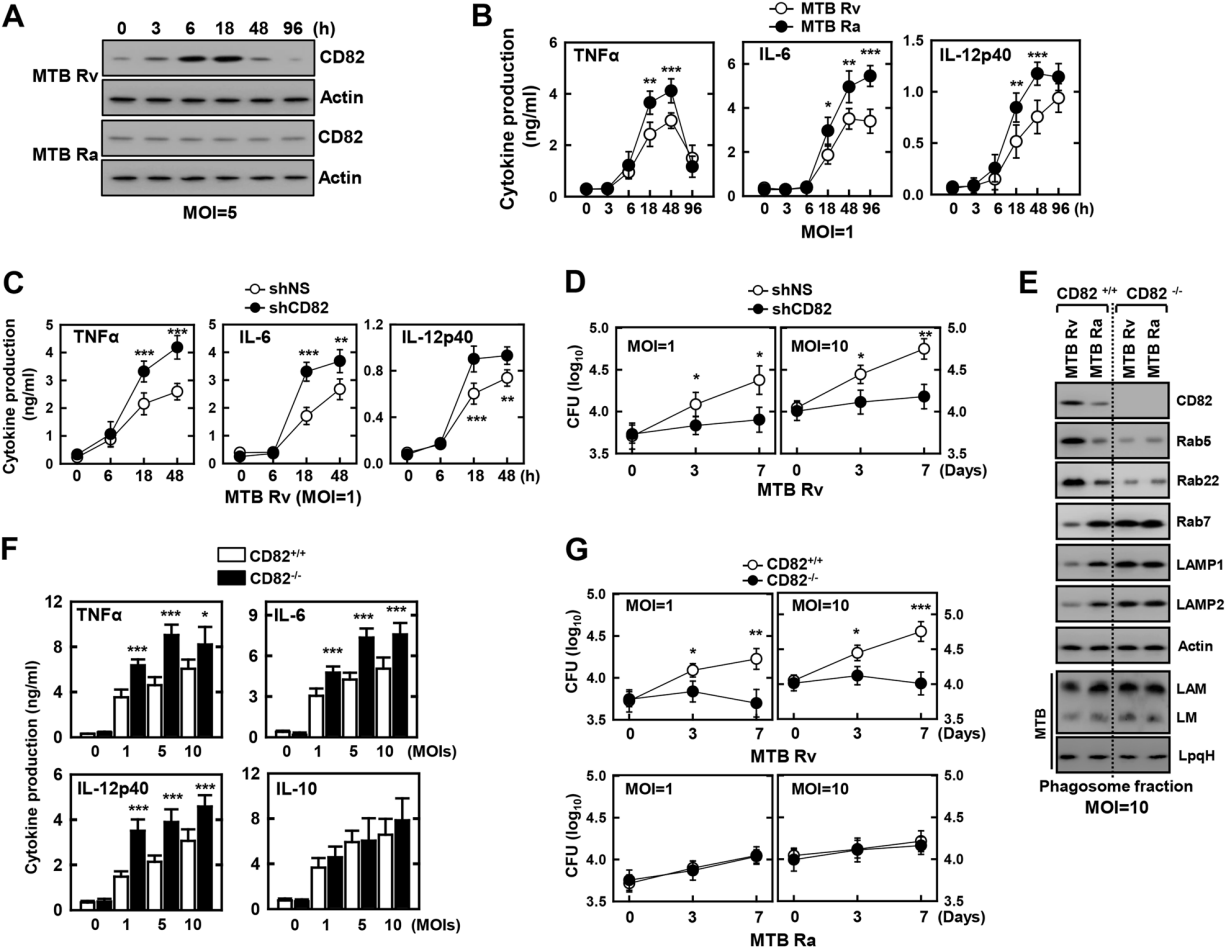
The effects of CD82 on cytokine production and bacterial survival in macrophages. BMDMs were infected with MTB Rv or MTB Ra for the indicated times, followed by **a** immunoblotting (IB) with αCD82 and αActin, or **b** culture supernatants were harvested and analyzed for cytokine ELISA for TNFα, IL-6, and IL-12p40. BMDMs were transduced with lentivirus-shRNA-NS or lentivirus-shRNA-CD82 for 2 days (**c**, **d**) or BMDMs from CD82^+/+^ and CD82^−/−^ (**f**, **g**) were with MTB Rv or MTB Ra for the indicated times, followed by intracellular survival of MTB assessed by CFU assay (**c**, **f**), or culture supernatants were harvested at 18 h and analyzed for cytokine ELISA for TNFα, IL-6, and IL-12p40 (**d**, **g**). **e** BMDMs from CD82^+/+^ and CD82^−/−^ were infected with MTB Rv or MTB Ra for 6 h. Mycobacteria-containing phagosome fractions were subsequently purified by sucrose-step-gradient ultra-centrifugation, followed by IB to detect αCD82, αRab5, αRab22, αRab7, αLAMP1, αLAMP2, and αActin. The polyclonal MTB Ab and LpqH Ab detect the lipoglycans (LAM and LM) and lipoproteins (LpqH), respectively. The data are representative of four independent experiments with similar results (**a**, **e**). Data shown are the means ± SD of six experiments (**b**–**d**, **f**, **g**). Significant differences (*P *<* 0.05; **P *<* 0.01; ***P *<* 0.001) compared with shRNA-NS or CD82^+/+^ (Student’s *t-*test with Bonferroni adjustment). CFU colony-forming units

Consistent with the RNA-seq data, CD82 protein expression was significantly attenuated in MTB Ra-infected macrophages when compared with MTB Rv-infected macrophages (Fig. 2a and S2A). We examined the relationship between mycobacterial virulence and inflammation. As previously reported^22,23^, macrophages infected with MTB Ra showed higher levels of proinflammatory cytokine production than those infected with MTB Rv (Fig. 2b).

We next examined the effects of CD82 on inflammatory responses and the intracellular growth of various mycobacteria. CD82 expression decreased production of proinflammatory cytokines and increased intracellular survival of mycobacteria in J774A.1 cells (Fig. S2B and C). Conversely, depletion of CD82 expression in macrophages of CD82-knockdown or CD82^−/−^ mice resulted in enhanced inflammation and reduced MTB growth (Fig. 2c–g, S2D and S3).

CD82 is actively recruited to phagosomes containing pathogenic fungi (C*ryptococcus neoformans*, *Candida albicans*, and *Aspergillus fumigatus*) and bacteria (*Escherichia coli* and *Staphylococcus aureus*) independent of Toll-like receptor signaling and is specifically recruited to pathogen-containing phagosomes prior to fusion with lysosomes^5,6^. MTB preferentially infects macrophages, although mycobacteria allow only early endosome membrane fusion and induce phagosome arrest by selective Rab GTPase recruitment to avoid fusion with late endosomes and lysosomes^26,35,36^. To investigate the subcellular localization and role of CD82, we examined the protein levels of Rab5, Rab22, Rab7, LAMP1, and LAMP2 (regulators of phagosomal maturation) in mycobacteria-containing phagosome fractions (phagosomes and phago-lysosomes) purified by sucrose step-gradient ultra-centrifugation. Consistent with the findings shown in Figs. 1 and 2a, MTB Rv-induced CD82 expression and recruitment of the early endosome markers Rab5 and Rab22 was enhanced in phagosomes of WT macrophages compared with those infected with MTB Ra. However, MTB Rv-containing phagosomes were recruited to late endosome and lysosome markers Rab7, LAMP1, and LAMP2 in CD82^−/−^ macrophages, indicating that mycobacterial phagosome–lysosome fusion was regulated in a CD82-dependent manner (Fig. 2e). Taken together, these data indicate that MTB Rv-induced CD82 expression and phagosome recruitment are essential for mycobacterial survival in macrophages.

### CD82 interaction with Rab5 and Rab22

**Fig. 3:**
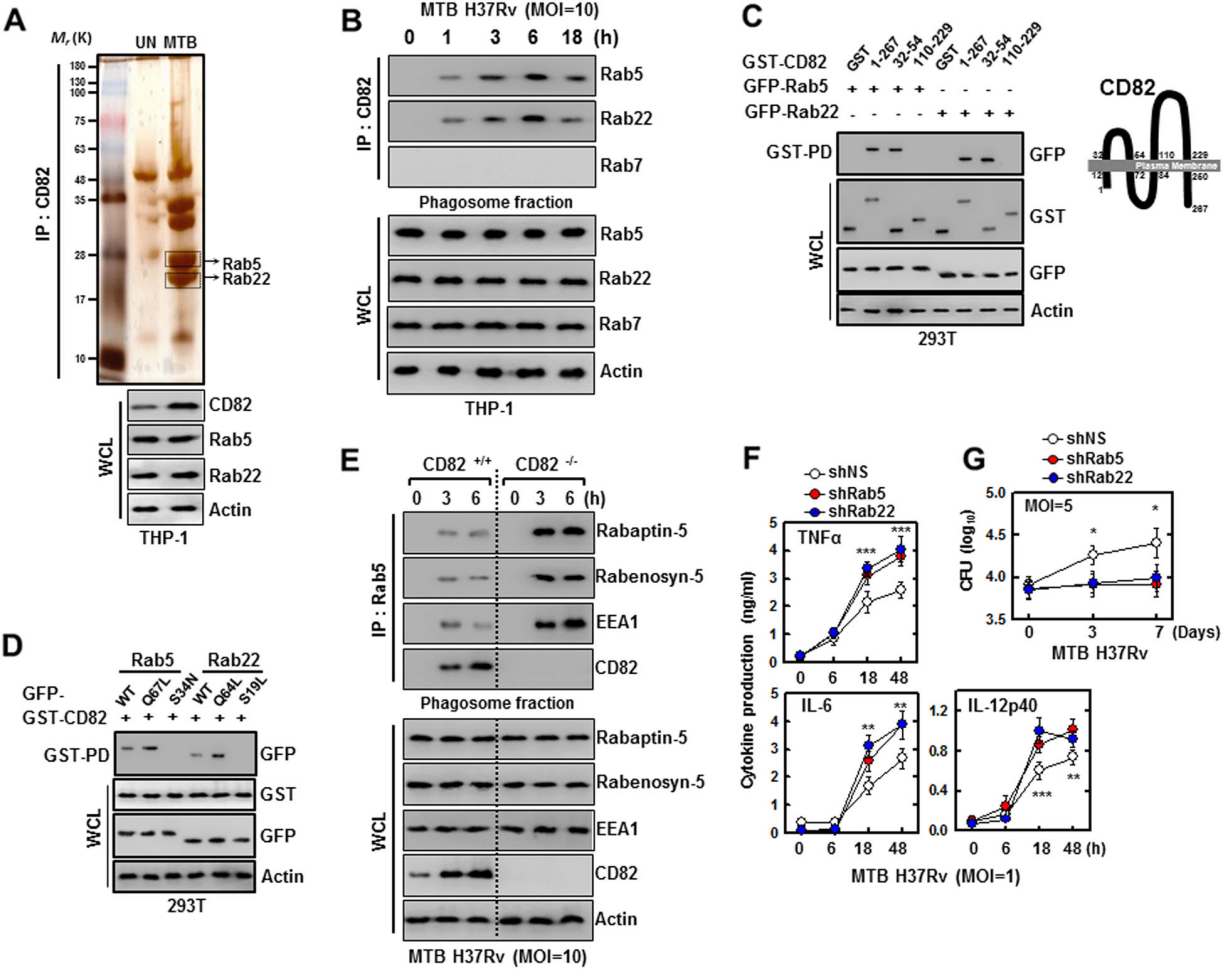
CD82 interaction with Rab5 and Rab22 leads to bock maturation. **a** Identification of Rab5 and Rab22 by mass spectrometry analysis in THP-1 cells were infected with MTB Rv or MTB Ra (MOI = 5) for 3 h. Whole cell lysates (WCLs) were used for IB with αCD82, αRab5, αRab22, and αActin. **b**, **e** THP-1 cells (**b**) and BMDMs (**e**) from CD82^+/+^ and CD82^−/−^ were infected with MTB Rv for the indicated times. Mycobacteria-containing phagosome fractions were subsequently purified by sucrose-step-gradient-ultra-centrifugation, followed by immunoprecipitation (IP) with αCD82 (**b**) or αRab5 (**e**) and IB with αRab5, αRab22, αRab7 (**b**) or αRabaptin-5, αRabenosyn-5, αEEA1, αCD82 (**e**), and αActin. **c** Binding mapping. (Left) At 48 h post transfection with mammalian GST or GST-82 and truncated mutant constructs together with GFP-Rab5 or GFP-Rab22, 293T cells were used for GST pulldown, followed by IB with αGFP. WCLs were used for IB with αGST, αGFP or αActin. (Right) Schematic diagrams of the structure of CD82. **d** 293T cells were co-transfected with GST-CD82 with GFP-Rab5 or GFP-Rab22 and truncated mutant, and subjected to GST pulldown, followed by IB with αGFP. WCLs were used for IB with αGST, αGFP or αActin. **f** BMDMs were transduced with lentivirus-shRNA-NS or lentivirus-shRNA-Rab5 or Rab22 for 2 days and infected with MTB Rv for the indicated times. Culture supernatants were harvested and analyzed for cytokine ELISA for TNFα, IL-6, and IL-12p40 (**f**) or intracellular survival of MTB was assessed by CFU assay (**g**). The data are representative of four independent experiments with similar results (**a**–**e**). Data shown are the means ± SD of six experiments (**f**, **g**). Significant differences (*P *<* 0.05; **P *<* 0.01; ***P *<* 0.001) compared with shRNA-NS (Student’s *t-*test with ANOVA for multiple comparisons). CFU colony-forming units

To establish a role for CD82 in the MTB virulence mechanisms involved in controlling intracellular survival of pathogenic mycobacteria in macrophages, we investigated whether CD82 interacts with molecules known to be involved in TB pathogenesis. CD82 complexes were subjected to co-immunoprecipitation (co-IP) from THP-1 cells infected with MTB Rv. The purified CD82 complexes selectively retrieved several endogenous proteins, as identified by mass spectrometry analysis, including Rab5 (25 K) and Rab22 (21 K) (Fig. 3a, b, and S4A). In the MTB-containing phagosome fraction, CD82 interacted strongly, although transiently (3–6 h), with endogenous Rab5 and Rab22, but not with Rab7 after infection with MTB Rv, and vice versa (data not shown).

Structurally, CD82 contains two cytoplasmic, four transmembrane, and two extracellular domains (Fig. 3c)^2^. In 293T cells, detailed mapping using GST pulldown assays with WT or truncated mutants of GST–CD82 mammalian fusions showed that the extracellular domains (aa32–54) of CD82 had only minimal binding affinity for Rab5 or Rab22 compared with those of CD82 WT (Fig. 3c). Furthermore, CD82 only interacted with the WT and guanosine triphosphate (GTP)-bound forms (QL), not with the guanosine diphosphate (GDP)-bound forms (SN or SL) of Rab5 and Rab22 (Fig. 3d).

Rab5 and Rab22 compose a Rab22–Rabex-5–Rab5 signaling cascade^37^ in which activated Rab22 recruits Rabex-5 and guanosine nucleotide exchange factor (GEF) to promote GDP-to-GTP exchange on Rab5^38,39^. Activated Rab5 then recruits downstream effector proteins such as Rabaptin-5, Rabenosyn-5, and EEA1, which mediate diverse endosomal processes including vesicle fusion and membrane trafficking^36,40^. In addition, Rab22 regulates the formation of tubular recycling endosomes, which are necessary for endosome-to-plasma membrane recycling of internalized materials^41^. To determine whether CD82 binding to activated Rab5 and Rab22 prevents interactions with their direct downstream effectors, we analyzed the performances of CD82 constructs in competition assays and demonstrated that increased levels of CD82 diminished the interaction between Rab5 and Rabaptin-5 or Rabenosyn-5 in HEK293T cells (Fig. S4B). Furthermore, in the MTB-containing phagosome fraction, endogenous co-IP experiments showed that Rab5 interactions with Rabaptin-5, Rabenosyn-5, or EEA1 were markedly increased in CD82^−/−^ macrophages infected with MTB Rv (Fig. 3e).

We next examined the effects of Rab5 and Rab22 on inflammatory responses and the intracellular growth of MTB. Depletion of Rab5 and Rab22 in macrophages resulted in enhanced inflammation and reduced MTB growth (Fig. 3f, g). Together, these results indicate that CD82, through its extracellular domain, specifically and potently interacts with the active forms of two endosomal Rabs, blocking their binding interactions, their three downstream effectors and contributing to TB pathogenesis.

### MTB-induced CD82 expression is regulated by DNA hypomethylation

**Fig. 4:**
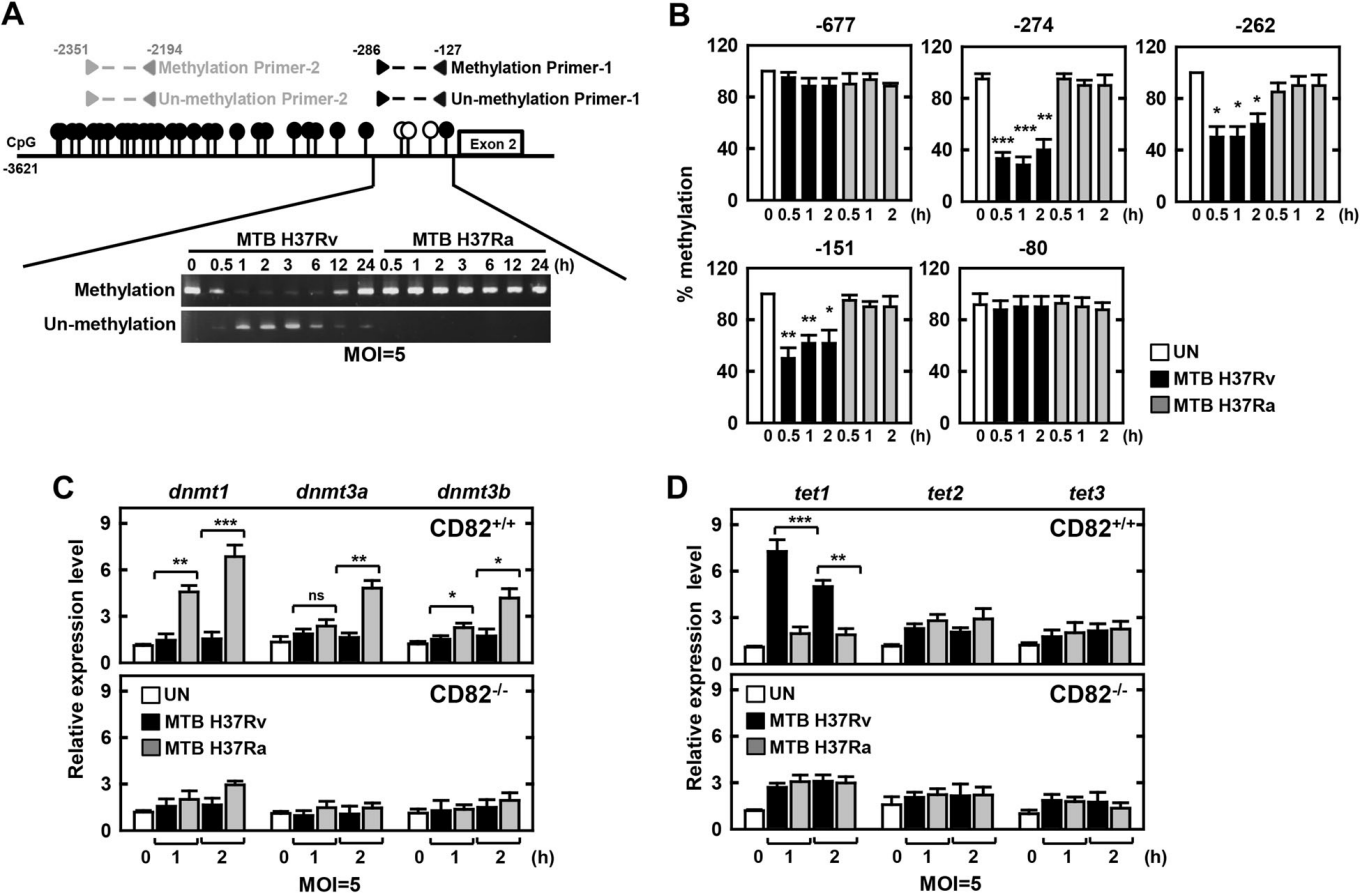
Methylation analysis of CD82 promoter region during MTB infection. **a** Schematic illustration of the CD82 promoter region. The CpG island map is indicated in the top panel, and the sequence amplified for methylation analysis is shown in the bottom panel. BMDMs were infected with MTB Rv or MTB Ra for the indicated times, followed by PCR with methylation or non-methylation primers. **b** Methylation status of CpG sites was measured by direct sodium bisulfate DNA sequencing as in the Supplementary Methods. Methylation status of −677, −274, −262, −151, or −80 regions was analyzed. At least 10 clones were sequenced for each group. **c**, **d** BMDMs from CD82^+/+^ and CD82^−/−^ were infected with MTB Rv or MTB Ra for the indicated times. Real-time qPCR analysis of dnmt or tet expression. The data are representative of five independent experiments with similar results (**a**). Data shown are the means ± SD of five experiments (**b**–**d**). Significant differences (*P *<* 0.05; **P *<* 0.01; ***P *<* 0.001) compared with CD82^+/+^ (Student’s *t-*test with Bonferroni adjustment)

MTB have evolved strategies to promote their survival by dramatically modifying the epigenetic mechanisms of the host cells they infect^42–45^. To effectively modulate gene expression within MTB-infected macrophages, MTBs must bring about epigenetic modifications at the appropriate genomic loci. We first investigated histone modification in macrophages using specific histone-targeted antibody-mediated upregulation of gene expression^43,44^. However, no changes in histone modification were observed during MTB Rv and MTB Ra infection (Fig. S5). Next, we examined whether CD82 DNA methylation directly influenced MTB-induced CD82 expression. Portions of the CD82 gene, spanning positions −127 to −286 bp or −2194 to −2351 bp, were analyzed with MethPrimer, which is a CpG island prediction program (http://www.urogene.org/methprimer/). The results showed that this fragment contained several CpG-rich regions (Fig. 4a, upper). Bisulfite modification followed by methylation-specific PCR allowed qualitative determination of methylation; macrophages infected with MTB Rv exhibited markedly lower CD82 methylation levels between positions −127 and −286 bp, which were maintained for up to 2 h longer than those infected with MTB Ra (Fig. 4a, lower). These findings were quantified by pyrosequencing analysis; we found that the regions at −151 bp, −262 bp, and −274 bp were significantly hypomethylated in macrophages infected with MTB Rv (Fig. 4b). Furthermore, we examined whether MTB Rv-induced CD82 expression was accompanied by sequential activation of the DNA methylation machinery (DNA methyltransferase (DNMT) and ten-eleven translocation methylcytosine dioxygenase (TET)). Interestingly, MTB Rv significantly increased expression patterns of DNMT isoforms, and TET1 expression was dependent on CD82 (Fig. 4c, d). Taken together, these results indicate that hypomethylation contributes to transcriptional regulation of CD82 through TET1 in macrophages.

### DNMT inhibition mediates inflammation and bacterial growth in a CD82-dependent manner

**Fig. 5:**
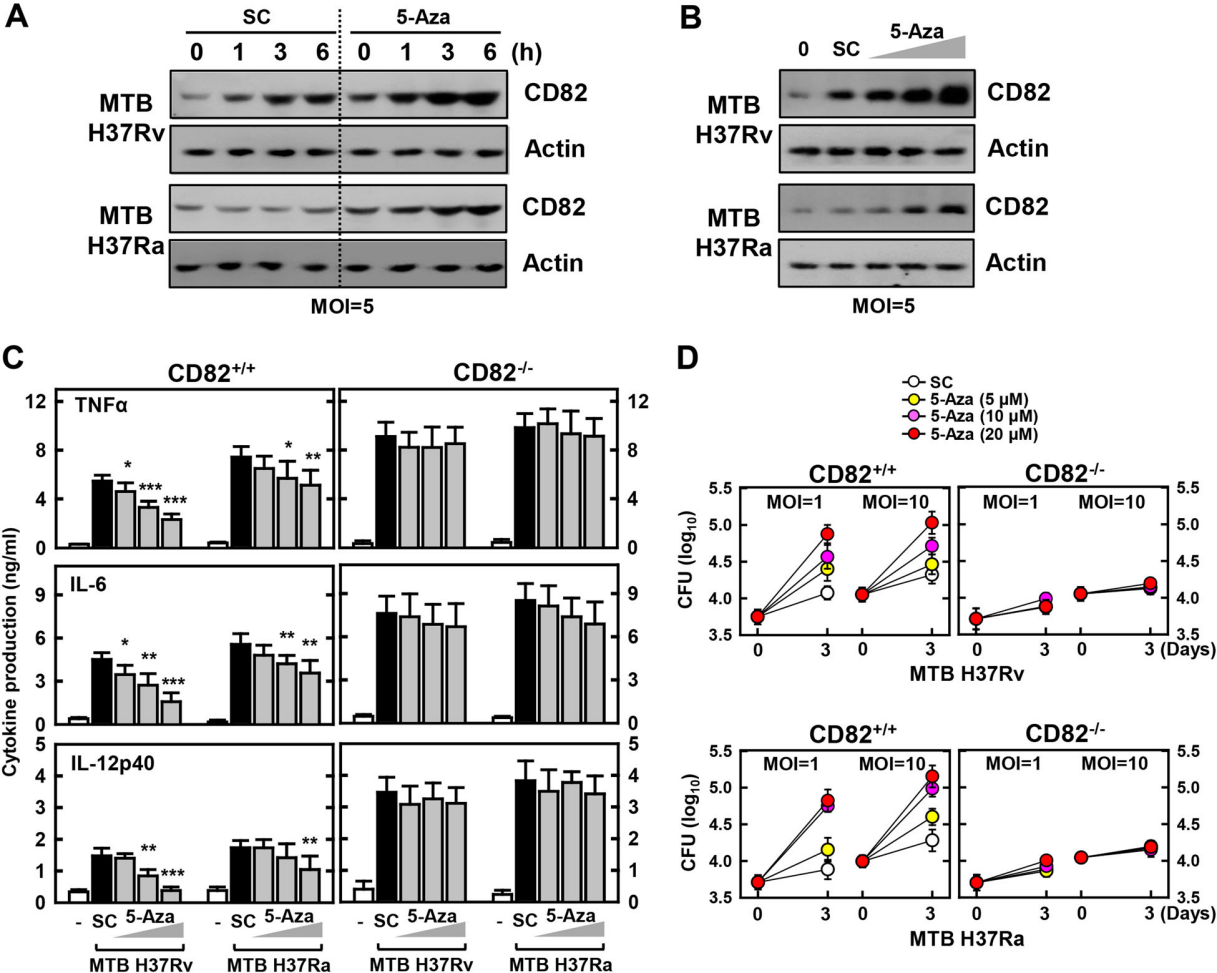
DNMT inhibition induces CD82 protein expression and anti-mycobacterial responses in a CD82-dependent manner. BMDMs were pretreated with 5-Aza (**a** for 10 μM and **b**–**d** for 5, 10, 20 μM) for 1 h and infected with MTB Rv or MTB Ra for the indicated times, followed by IB with αCD82 and αActin (**a**, **b**), culture supernatant harvesting at 18 h and analysis for cytokine ELISA for TNFα, IL-6, and IL-12p40 (**c**), or intracellular survival of MTB assessment by CFU assay (**d**). The data are representative of four independent experiments with similar results (**a**, **b**). Data shown are the means ± SD of five experiments (**c**, **d**). Significant differences (*P *<* 0.05; **P *<* 0.01; ***P *<* 0.001) compared with solvent control (Student’s *t-*test with Bonferroni adjustment). SC solvent control 0.1% DMSO, CFU colony-forming units

To further investigate the role of DNA methylation modification in CD82 expression and intracellular survival of MTB, CD82 and inflammatory cytokine expression levels were measured in the presence of a DNMT inhibitor (5-Aza-2′-deoxycytidine) in MTB-infected macrophages. CD82 expression was markedly increased by the dose-dependent inhibition of DNA methylation following infection with MTB, which was confirmed by western blotting (Fig. 5a, b). In addition, DNMT inhibitor treatment significantly reduced both the production of tumor necrosis factor-α (TNF-α), interleukin (IL)-6, and IL-12p40 and bacterial clearance in a CD82-dependent manner (Fig. 5c, d). These results demonstrate that DNA methylation participates in MTB-induced expression of CD82 and in the CD82-mediated intracellular survival of MTB.

### CD82 is a direct target of RUNX1

**Fig. 6:**
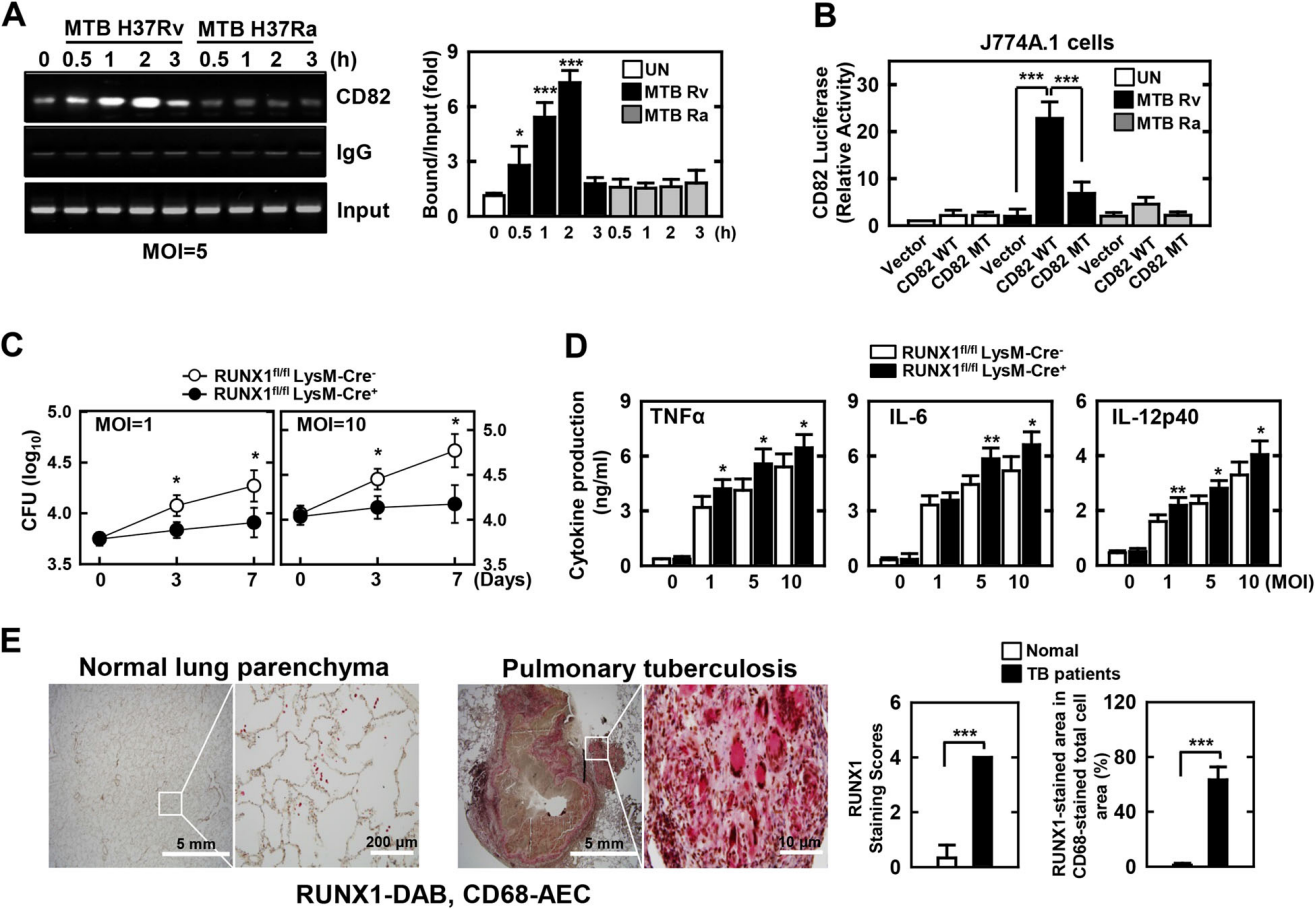
CD82 associates with RUNX1. **a** ChIP analyses of RUNX1. (Left) BMDMs were infected with MTB Rv for the indicated times, and subjected to semi-PCR analysis using primers specific for CD82. The data are representative of four independent experiments with similar results. (Right) The densitometry results of all four independent RUNX1-CD82 ChIP assays. **b** J774A.1 cells were transfected with CD82-luciferase reporter constructs (CD82 WT or MT carrying point mutations in the critical RUNX1-binding site) and infected with MTB Rv (MOI = 5) for 6 h. The promoter activities were determined by luciferase assays and normalized to *Renilla* luciferase enzyme activities. **c**, **d** BMDMs from RUNX1^fl/fl^ LysM-Cre+ and RUNX1^fl/fl^ LysM-Cre- mice were infected with MTB Rv for the indicated times, followed by **c** intracellular survival of MTB assessment by CFU assay or **d** culture supernatant harvesting at 18 h and analysis by cytokine ELISA for TNFα, IL-6, and IL-12p40. **e** Immunohistochemical analysis to examine RUNX1-DAB and CD68-AEC expression levels in healthy controls and patients from pulmonary TB. Representative images from 5 independent healthy controls and patients are shown. Insets, enlargement of outlined areas. Data shown are the means ± SD of five experiments (**b**–**d**). Significant differences (*P *<* 0.05; **P *<* 0.01; ***P *<* 0.001) compared with MTB Ra or RUNX1^fl/fl^ LysM-Cre- (Student’s *t-*test with Bonferroni adjustment). CFU colony-forming units

Proximal and distal regulatory elements, including enhancers, play a critical role in regulating gene activity. Although transcription factor binding to these elements correlates with hypomethylated regions in CD82 expression, this interaction is poorly understood^46,47^. To identify the mechanism(s) underlying the role of CD82 in MTB infection, we used computational transcription factor binding site analysis^48,49^, which predicted the binding of one transcription factor—RUNX1—within the CD82 hypomethylation region. Nine putative RUNX1 consensus motifs were identified in the promoter region of CD82 (Fig. S6A). Thus, we considered whether the MTB-induced CD82 expression pathway was involved in the activation of RUNX1, performing chromatin immunoprecipitation (ChIP) assays to assess the binding of RUNX1 to promoters of CD82. We found a strong interaction between RUNX1 and CD82 promoter regions in BMDMs after MTB Rv infection (Fig. 6a). We next determined whether differential RUNX1 occupancy among the RUNX1 sites in the CD82 promoters correlated with significant differences in promoter activities. We performed cell-based Luciferase (Luc) reporter assays using a 2 kb DNA fragment of the promoters of CD82 (Fig. 6b). MTB Rv-induced CD82 significantly increased the activity of WT promoter constructs (approximately 2 kb) of CD82 in J774A.1 cells. Mutation of RUNX1 consensus motifs within the CD82 promoters significantly decreased reporter gene activity, and CD82 expression was abrogated in MTB Rv-infected RUNX1-deficient BMDMs (Fig. 6b and S6B). Furthermore, MTB Rv-induced CD82 and recruitment of the early endosome markers Rab5 and Rab22 was enhanced in phagosomes of WT macrophages infected with MTB Rv when compared with those infected with MTB Ra. However, MTB Rv-containing phagosomes were recruited to late endosomal and lysosomal markers Rab7, LAMP1, and LAMP2 in RUNX1-deficient BMDMs, indicating that mycobacterial phagosome–lysosome fusion was regulated by the binding of RUNX1 to hypomethylated CD82 (Fig. S6C).

We next examined the effects of RUNX1 on inflammatory responses and intracellular growth of various mycobacteria. Depletion of RUNX1 expression in macrophages led to enhanced inflammation and reduced MTB growth (Fig. 6c, d). Interestingly, when we examined pulmonary RUNX1 expression between healthy controls and patients with pulmonary TB, we found weak positivity in the nuclei of bronchiole epithelial cells, pneumocytes, and alveolar macrophages in blood vessels of normal lung parenchyma, whereas cases of pulmonary TB exhibited strong positivity in lymphocytes, epithelioid cells, and multinucleated giant cells in granulomas (Fig. 6e). Taken together, these results show that virulent MTB-induced hypomethylated CD82 interacts with RUNX1 and contributes to an MTB virulence mechanism of TB pathogenesis and that RUNX1 therefore has clinical significance in human TB.

### CD82 and RUNX1 contribute to TB pathogenesis in vivo

**Fig. 7:**
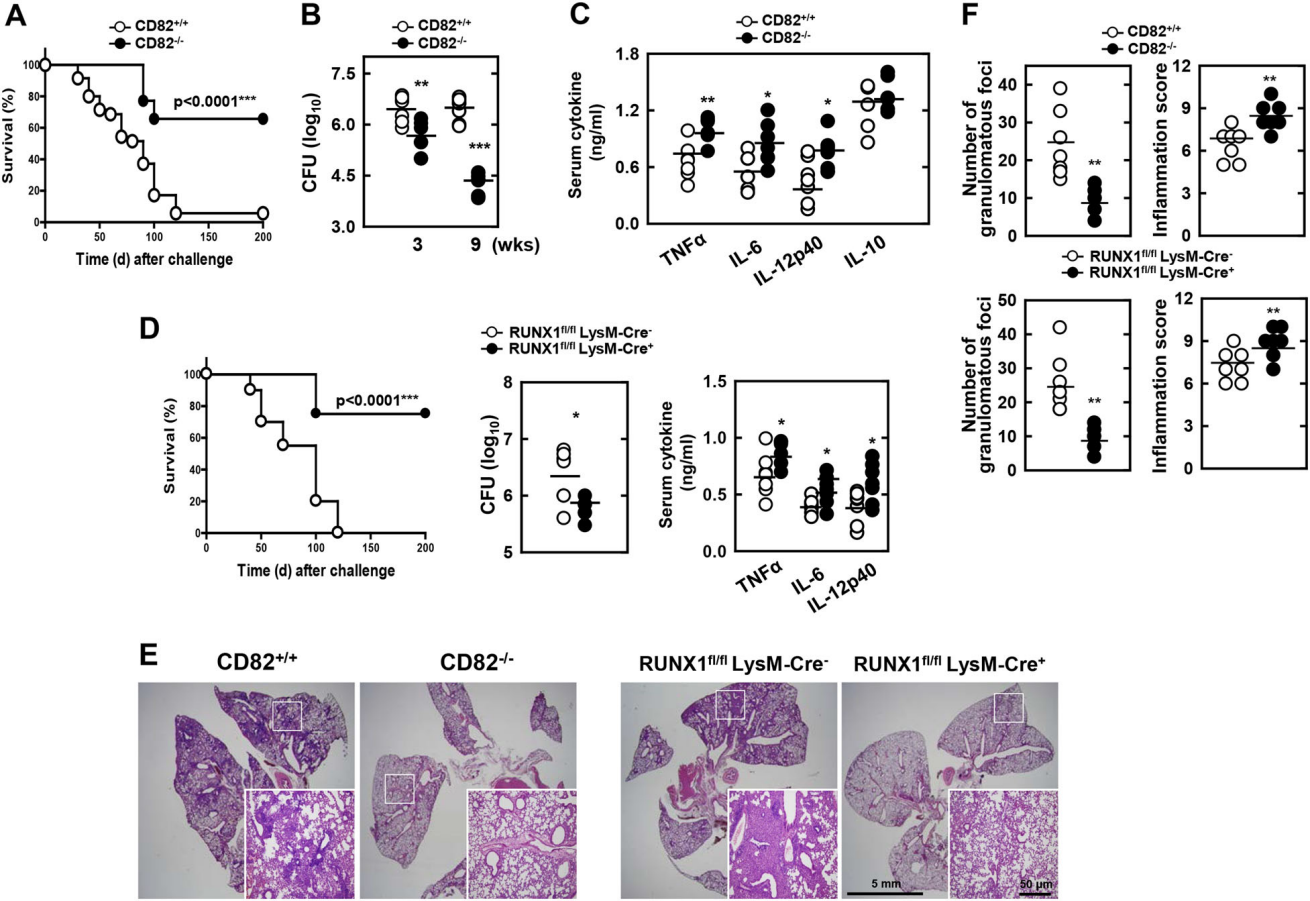
Modulation of CD82 and RUNX1 affects TB pathogenesis after MTB infection. **a**, **d** Survival of (**a**) CD82^+/+^ and CD82^−/−^ mice (*n* = 35) or (**d**) RUNX1^fl/fl^ LysM-Cre+ and RUNX1^fl/fl^ LysM-Cre- mice (*n* = 20) infected with a high intravenous dose (1 × 10^8^ CFUs per mouse) of MTB Rv and monitored for 200 days. Significant differences in comparison to the control mice are indicated (log-rank test). **b**–**f** CD82^+/+^ and CD82^−/−^ or RUNX1^fl/fl^ LysM-Cre+ and RUNX1^fl/fl^ LysM-Cre- mice were infected with an intranasal dose (1 × 10^3^ CFUs per mouse) of MTB Rv. **b**, **d** Bacterial loads in lungs and **c**, **d** serum cytokine levels were determined at 3 weeks; *n* = 7. **e** Histopathology scores were obtained from H&E-stained lung sections at 3 weeks, as described in the Methods. Insets, enlargement of outlined areas. **f** Number of granulomas and inflammation scores observed in seven different lung sections per mouse. Black bars represent the median values. Student’s *t*-test and Grubbs’ outlier test were used for statistical analysis. The data are representative of two independent experiments with similar results. Significant differences (*P *<* 0.05; **P *<* 0.01; ***P *<* 0.001) compared with CD82^+/+^ and RUNX1^fl/fl^ LysM-Cre- (Student’s *t-*test with Bonferroni adjustment). CFU colony-forming units

Drawing on the observation that CD82 associates with RUNX1 and Rab5/22, and contributes to MTB virulence in macrophages (Figs. 2 and 6), we next evaluated CD82–RUNX1-mediated TB pathogenesis in a mouse model of established TB in vivo^27,50^. After infection with a high intravenous dose of MTB Rv, CD82^−/−^ mice showed an increased survival rate (median survival, 90 days) compared with CD82^+/+^ mice (median survival, 70 days) (Fig. 7a). To determine whether these effects were due to impaired bacterial clearance in CD82^−/−^ mice, we measured bacterial loads and serum cytokine levels. We found significantly reduced bacillary loads in the lungs and enhanced inflammation in sera of CD82^−/−^ mice (Fig. 7b, c). Furthermore, RUNX1-deficient mice also exhibited significantly increased survival rates, decreased bacterial loads in the lungs, and elevated inflammation in sera (Fig. 7d). Consistent with mycobacteria loads and inflammation levels, CD82^−/−^ and RUNX1-deficient mice showed a decreased incidence of pathological hallmarks, including size and number of lung granulomatous lesions and increased inflammation scores in the lungs when compared with WT mice (Fig. 7e, f and S7). These results unambiguously show that MTB virulence is substantially affected by CD82 and RUNX1.

## Discussion

The central finding of this study is that MTB Rv-induced hypomethylated CD82 interacts with RUNX1 and Rab5/22 and contributes to TB pathogenesis (Fig. S8). Specifically, we found that: (i) virulent MTB-specific hypomethylation in the promoter and the expression of CD82 in vitro and in vivo was functionally required for RUNX1 and Rab5/22 interactions with CD82 in macrophages; (ii) several promoter regions (e.g., −151 bp, −262 bp, and −274 bp) of CD82 were hypomethylated by MTB Rv in a TET1-specific manner; (iii) hypomethylated CD82 was sufficient for interaction with RUNX1 in the nucleus and for subsequent antimicrobial activity through CD82 protein expression-mediated inflammation inhibition; (iv) the N-terminal aa32–54 of CD82 were sufficient for interaction with the GTP-bound forms of Rab5 and Rab22 in phagosomes, leading to the blockage of their binding interactions with their three downstream effectors and to phagosome–lysosome biogenesis and subsequent antimicrobial activity; (v) CD82 actions in the chromosome and phagosome are functionally and genetically separable, and CD82 thus functions in a localization-specific binding-dependent manner; (vi) CD82- and RUNX1-dependent TB pathogenesis in MTB infection occurs in vivo; and (vii) levels of CD82 and RUNX1 were elevated in granulomas from pulmonary TB patients, indicating the clinical significance of CD82 in human TB. Collectively, these observations indicate that the CD82–RUNX1–Rab5/22 axis may be an MTB virulence mechanism of TB pathogenesis. Altered CD82–RUNX1 gene expression may also have clinical implications for other infection-associated/inflammation-associated conditions, creating novel venues for translational research, such as in the expanding field of host-targeted therapy for infectious diseases.

Although it is well established that the expression of host genes is affected by epigenetic modification during MTB infection^43–45^, new candidates for further development of effective therapeutics against MTB infection are needed^15,16^. Recent reports have shown that the NLRP3 promoter region from −700 to −500 bp is demethylated and NLRP3 is subsequently expressed after MTB infection, supporting the hypothesis that MTB targets host NLRP3 for antibacterial host responses via ESAT-6 to activate the NLRP3 inflammasome^26,45^. MTB reprograms the host epigenome directly through the binding of the mycobacterial-secreted DNA methyltransferase Rv2966c virulence factor to host histones H3 and H4 and the subsequent induction of non-CpG methylation, or indirectly through histone hypoacetylation and the suppression of CIITA transcription by MTB 19 kDa lipoprotein to prevent antigen processing and MHC-II expression^43,51^. Furthermore, MTB-secreted DNA methyltransferase Rv1988, which acts directly at the level of host chromatin, methylates histone H3 at a non-canonical arginine residue (H3R42) present within its core-structured region, represses immunologically important genes, and adversely affects the health of infected mice^44^. In this study, we provided evidence that CD82 expression promoted by MTB Rv is regulated by DNA methylation in vivo; however, future studies will be needed to clarify the MTB virulence proteins that are involved in the MTB-induced modification of CD82 expression. This study not only revealed the epigenetic mechanisms of CD82-mediated MTB virulence, but also provided new insights into the regulatory mechanisms underlying MTB infection and the interactions between host and pathogen.

Several mechanisms are involved in controlling intracellular survival of pathogenic mycobacteria, but the regulation of these mechanisms remains poorly understood. Several groups have shown that heme oxygenase-1 (HO1) is induced shortly after MTB infection in mice and in human TB macrophages^52–54^, where it colocalizes within infected cells. HO1 also mediates proinflammatory cytokine production in human macrophages while creating a permissive environment for intracellular bacterial replication. Furthermore, Awuh et al.^55^ showed that kelch-like ECH-associated protein 1 (Keap1), an oxidative stress sensor, regulates inflammatory signaling and the intracellular survival of *Mycobacterium avium* in human primary macrophages. They presented evidence of a mechanism whereby the Keap1/Cul3-Rbx E3 ubiquitin ligase complex regulates IKKβ activity through ubiquitination and degradation and showed that accumulation of p-IKKβ results in increased translocation of the transcription factors NF-κB, IRF1, and IRF5, as well as production of target gene expression in Keap1-knockdown macrophages. They also observed Keap1 regulation of TBK1 in *M. avium*-infected macrophages, as evidenced by increases in TBK1 and p-TBK1 after Keap1 knockdown. Here, we suggested a novel role for CD82 in regulating inflammatory signaling and the intracellular survival of MTB during infection in murine and in human TB lesions. Future studies will be necessary to determine whether CD82 is beneficial or detrimental during human TB pathogenesis.

The ubiquitously expressed tetraspanin CD82 restrains cell migration and cell invasion by modulating both cell–matrix and cell–cell adhesion, and by confining outside–in pro-motility signaling. This restraint at the least contributes to, if not determines, metastasis-suppressive activity and, also likely, the physiological functions of CD82^56–59^. Palmitoylated CD82 specifically recruits interaction partners, including epidermal growth factor receptor, EWI-2, integrin α6, c-Met, and Vangl1, to signaling platforms such as lipid rafts and glycosynapses^56,60,61^. Furthermore, CD82 interacts with: (i) p12CDK2-AP1 to regulate the proliferation and survival of human oral squamous cell carcinoma cells^57^, (ii) DARC to maintain quiescence in long-term repopulation of hematopoietic stem cells^58^, and (iii) tissue inhibitor of metalloproteinases-1 (TIMP-1), where the proteins colocalize in both pancreatic ductal adenocarcinoma cell lines and clinical samples. Moreover, CD82 facilitates membrane-bound TIMP-1 endocytosis, which significantly contributes to the anti-migration effects of TIMP-1^59^. Here, we identified CD82 as a novel virulence factor of TB pathogenesis. We showed that virulent MTB induced host cell CD82 expression and bound to RUNX1 in the nucleus and Rab5/22 in the phagosome.

The RUNX1 transcription factor plays important roles as follows: (i) in tissue homeostasis through its effects on hematopoiesis and hematopoietic function and is a tumor suppressor gene in the murine gastrointestinal tract^62^, (ii) RUNX1-deficient afferents impair visceral nociception, exacerbating the phenotypes in dextran sodium sulfate-induced colitis^63^, and (iii) RUNX1 functions as a cytoplasmic attenuator of nuclear factor (NF)-κB signaling in the repression in hematopoietic cells of myeloid tumors by inhibition of the kinase activity of IKK through physical interaction between RUNX1 and IKK. Remarkably, aberrant activation of NF-κB signaling is induced not only by targeted disruption but also by the leukemia-related gene alteration of RUNX1^64^. Our results partly correlate with those of a previous study showing that RUNX1 binds to CD82 in macrophages and suppresses MTB-induced infection and inflammation. Furthermore, phagosome–lysosome vesicle fusion/trafficking is targeted by MTB-induced CD82. Here, we showed that CD82 specifically and potently interacts with the endosomally activated forms of Rab5 and Rab22, blocking their interactions with their three downstream effectors (Rabaptin-5, Rabenosyn-5, and EEA1). The actions of CD82 in the chromosome and phagosome are functionally and genetically separable; CD82 thus operates in a localization-specific, binding-dependent manner. In this study, CD82 interacted with a number of host cell proteins, including enzymes, and a broad spectrum of structural and functional subcellular organellar proteins, revealing a new facet of the role of CD82 in the regulation of innate host infection and immune responses.

In conclusion, we provide evidence of a critical role of a hypomethylated CD82-mediated interaction between RUNX1 and Rab5/22 that contributes to MTB virulence (Fig. S8). These observations reveal a new role for CD82 in regulating infection and immunity against MTB; however, a detailed understanding of their role in MTB infection is still needed. This study contributes to our understanding of TB biology and to the development of new therapeutic approaches to help address the worldwide TB pandemic. Further study is required to examine the diverse array of functions of CD82 and RUNX1, the complexity of the regulation of CD82–RUNX1–Rab5/22, and the prospects for targeting CD82 as a therapeutic approach for the treatment of various infectious diseases.

## Accession number

The GenBank accession number for the CD82 gene is NM_001136055.2.

## Supplemental Information

Supplemental Information includes figures, supplemental experimental procedures, and supplemental references.

## Electronic supplementary material

Supplemental Information
