# Supplementary material for: CD82 hypomethylation is essential for tuberculosis pathogenesis via regulation of RUNX1-Rab5/22
Source: Exp Mol Med. 2018 May 14;50(5):62. doi: 10.1038/s12276-018-0091-4 (PMC5951854; doi:10.1038/s12276-018-0091-4)
Supplement: Supplementary file 1 — Supplemental Information [file 12276_2018_91_MOESM1_ESM.doc]

**Supplemental Information**

**
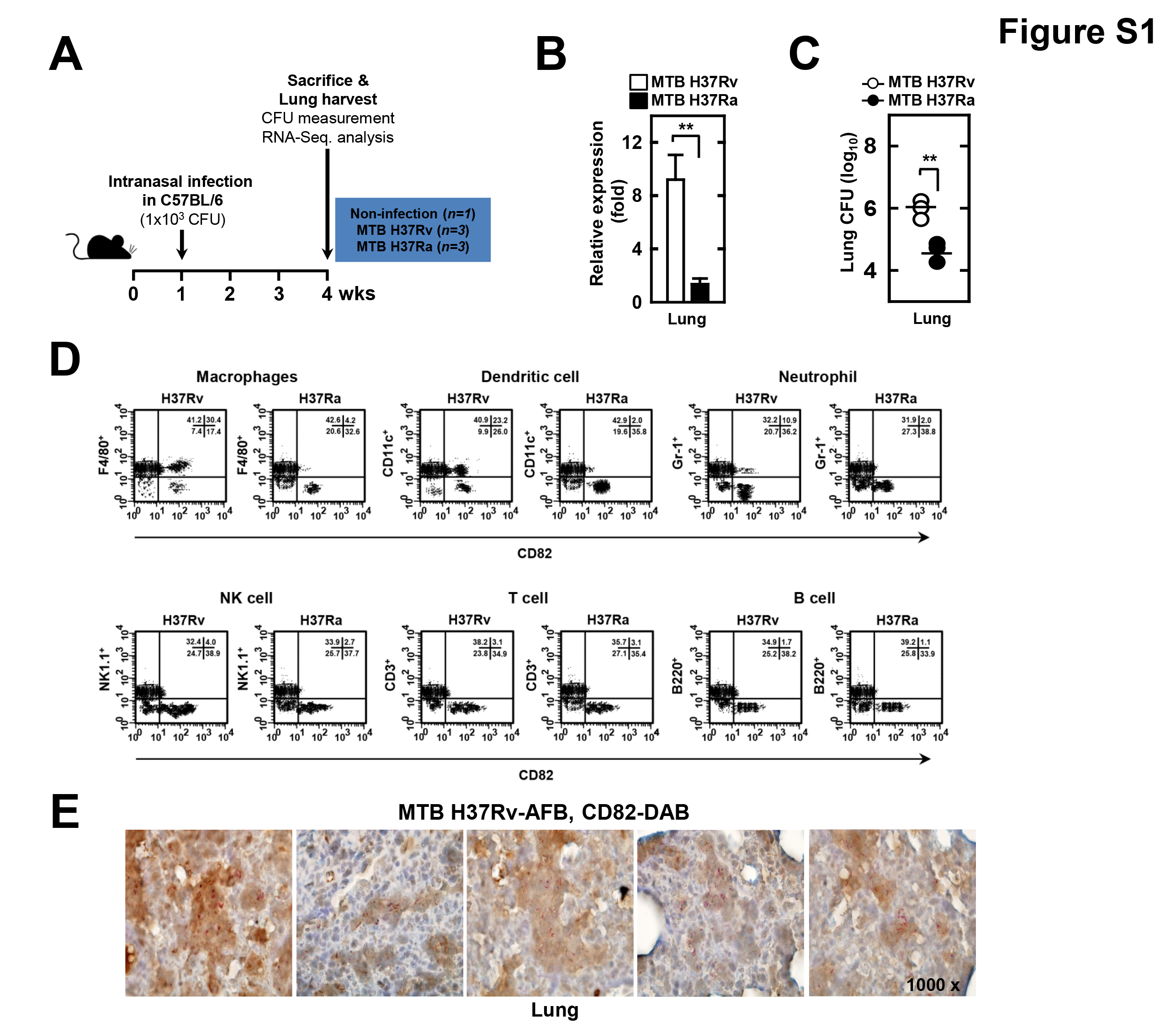
**

**Figure S1. Identification of MTB Rv-specific CD82 restrict macrophage infiltration.**

(**A**) Schematic design of the mycobacteria-infected TB model and RNA-seq. analysis. (**B**) Real-time qPCR analysis of CD82 expression or (**C**) Bacterial loads in lung from MTB Rv- or MTB Ra-infected mice. Biological replicates (*n*=3) for each condition were performed (**A**-**C**). Significant differences (***P* < 0.01) compared with both groups. (**D**) Representative dot plot gated on the macrophage marker F4/80+, dendritic cell marker CD11c+, neutrophil marker Gr-1+, NK cell marker NK1.1+, T cell marker CD3+, or B cell marker B220+ population cells stained for the CD82+ on the lung cell lysates. The representative dot plots of three independent experiments. The single-cell suspension was generated by the Dispase method [^1^](#_ENREF_1). (E) Mycobacterial infection of the lung using Mtb-infected macrophages. Bright-field images of fixed sections stained with AFB (purple), immunohistochemistry (anti-mouse CD82, DAB).

**
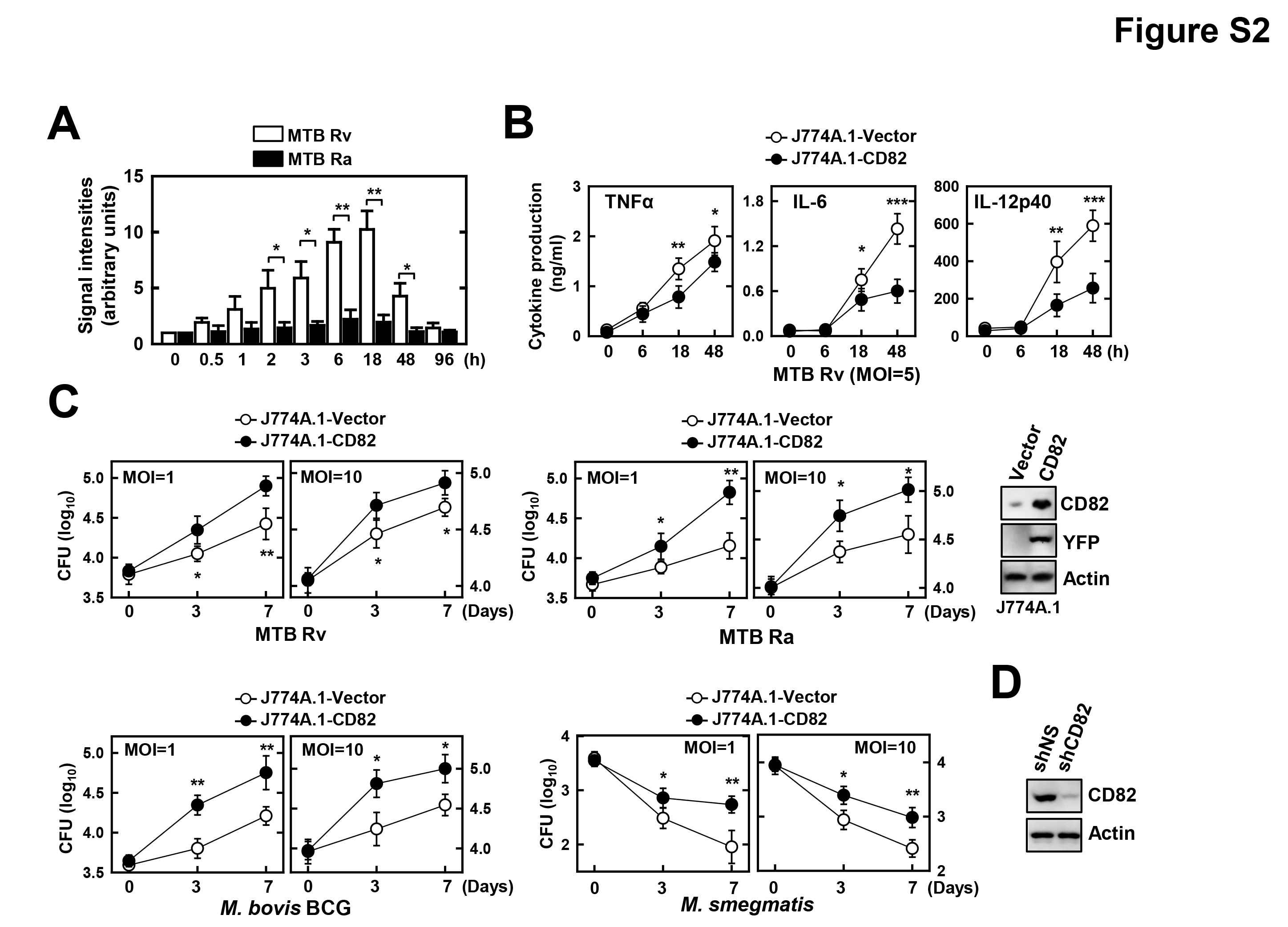
**

**Figure S2. CD82’s effect on inflammation and mycobacterial growth.** (**A**) Densitometry values of the CD82 were normalized to the Actin related to Fig. 2A. (**B**) J774A.1 cells containing vector or CD82 were infected with MTB Rv (MOI=5) for the indicated times, and culture supernatants were harvested and analyzed for cytokine ELISA for TNF-α, IL-6, and IL12p40. (**C**) Intracellular survival of MTB was assessed by CFU assay. J774A.1 cells containing vector or CD82 were infected with various mycobacteria (MOI=1 or 10) for the indicated times, and then lysed to determine intracellular bacterial loads. (**D**) BMDMs was transduced with lentivirus-shRNA-NS or lentivirus-shRNA-CD82 (MOI = 100) with polybrene (8 μg/mL) (right) for 2 days, followed by IB with αCD82, and αActin. The data are representative of five independent experiments with similar results (**D**). Data shown are the mean ± SD of five experiments (**A**-**C**). Significant differences (**P* < 0.05; ***P* < 0.01; ****P* < 0.001) compared with Vector. CFU, colony-forming units.

**
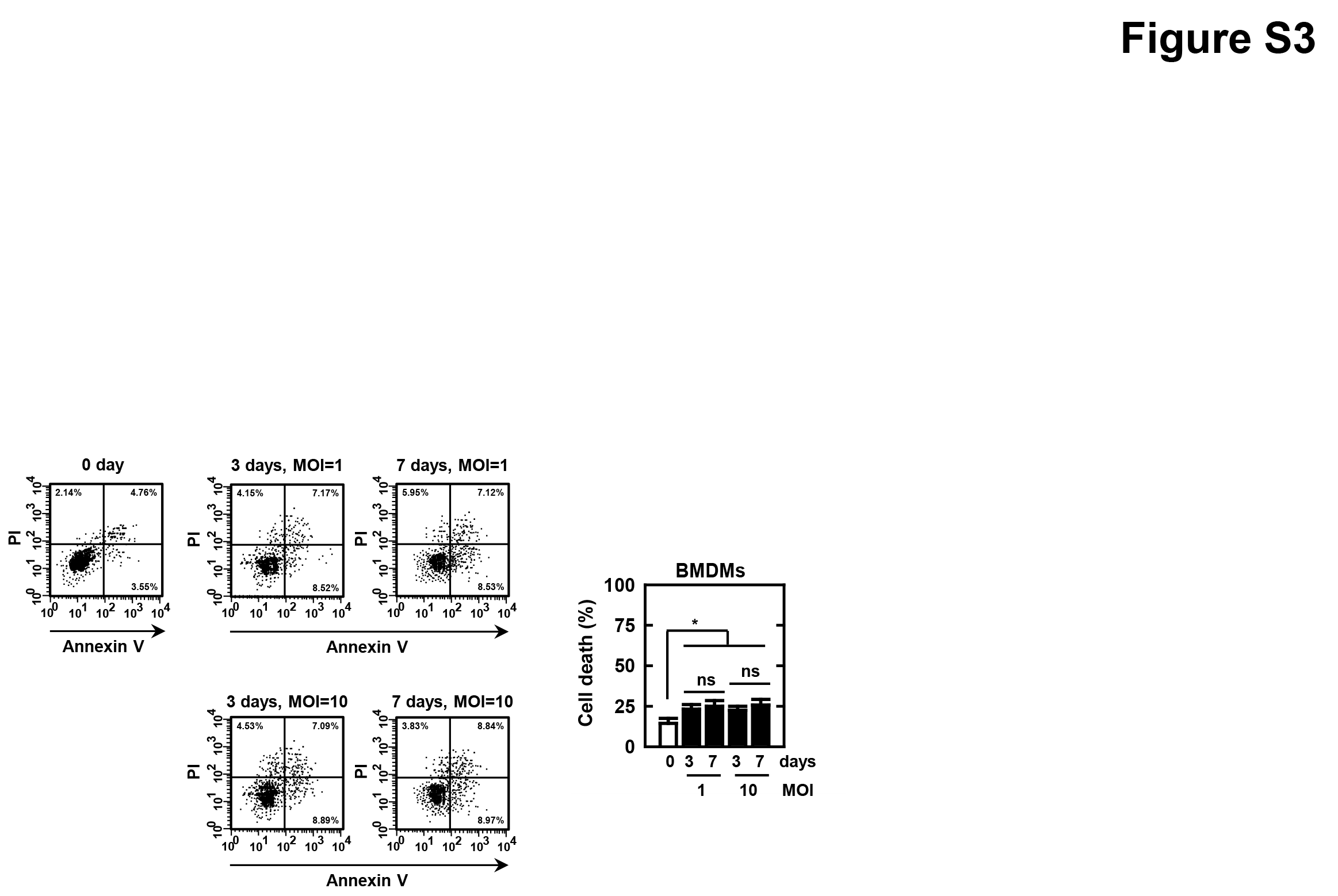
**

**Figure S3. The infection strategy was not toxic to macrophages cells.** The BMDMs were stained with annexin V and PI. The percentage of cells that are positive (annexin V- and PI-stained cells) in each quadrant is indicated. The results are representative of three experiments (left). Bar graph indicates the cell death (right). Significant differences (**P* < 0.05) compared with 0 day (un-infected cells). ns, no-significant.

**
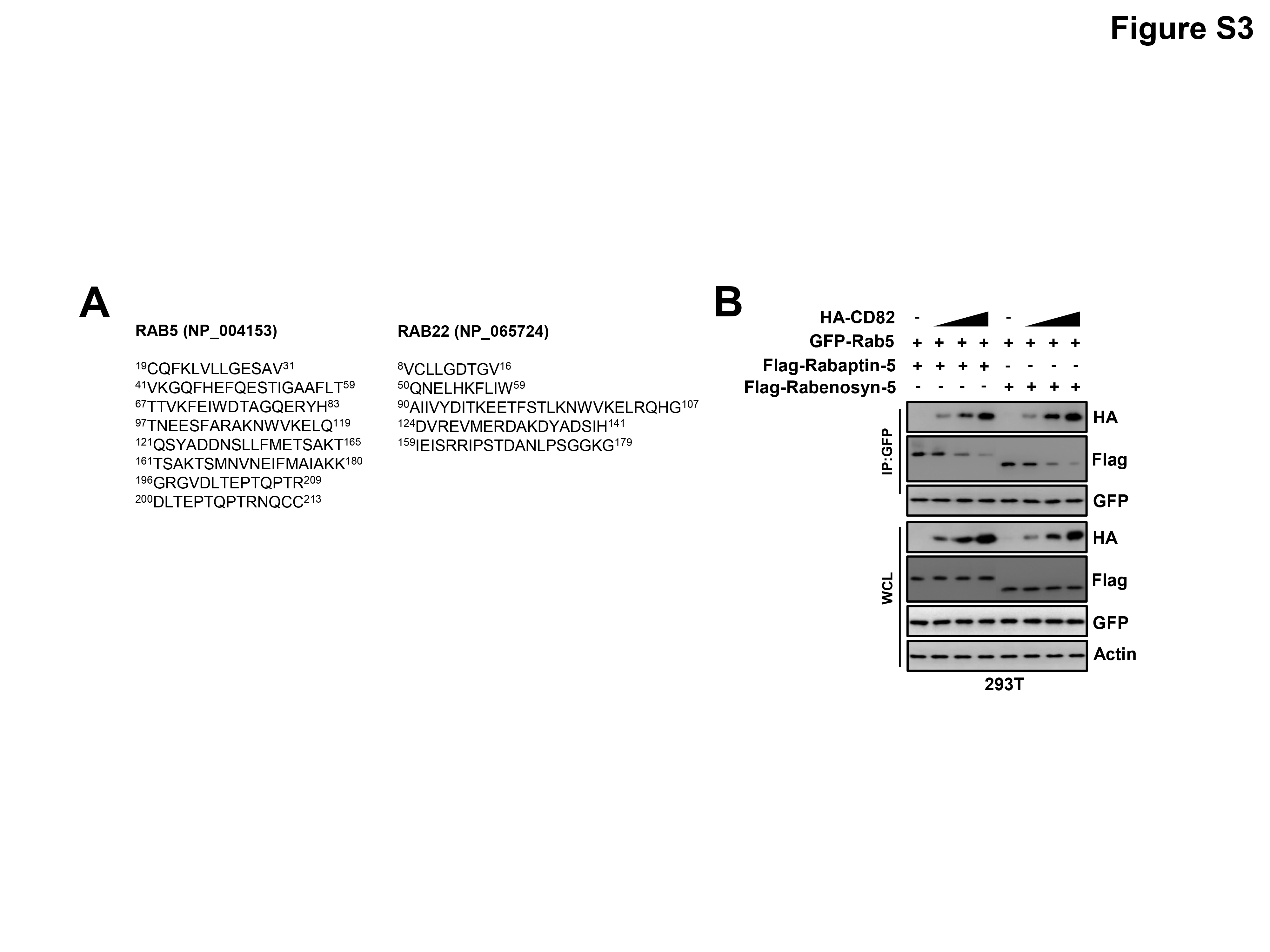
**

**Figure S4. CD82 interacts with Rab5 and blocking their binding interactions with the Rabaptin-5 or Rabenosyn-5.** (**A**) Identified of peptides by mass spectrometry analysis related to Fig. 3A. (**B**) At 48 hr post-transfection with mammalian GFP-Rab5 or HA-CD82 constructs together with Flag-Rabaptin-5 or Flag-Rabenosyn-5, followed by IP with αGFP and IB with αHA, αFlag, αGFP, and αActin. The data are representative of three independent experiments with similar results.

**
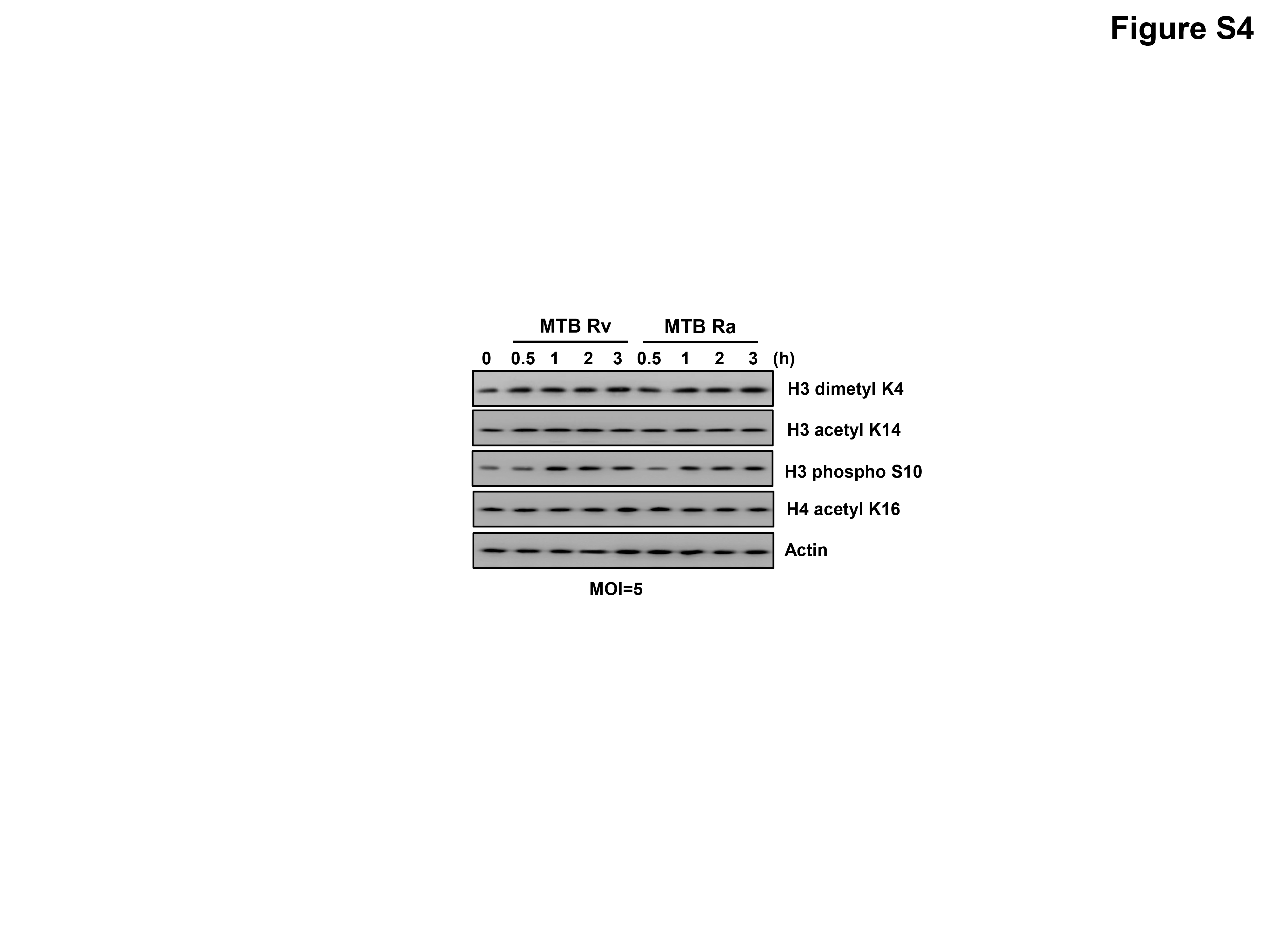
**

**Figure S5. The histone modification has no difference during MTB Rv and MTB Ra infection.** BMDMs were infected with MTB Rv or MTB Ra (MOI=5) for the indicated times, followed by IB with αHistone H3 dimetyl K4, αHistone H3 acetyl K14, αHistone phospho S10, αHistone H4 acetyl K16, and αActin. The data are representative of three independent experiments with similar results.

**
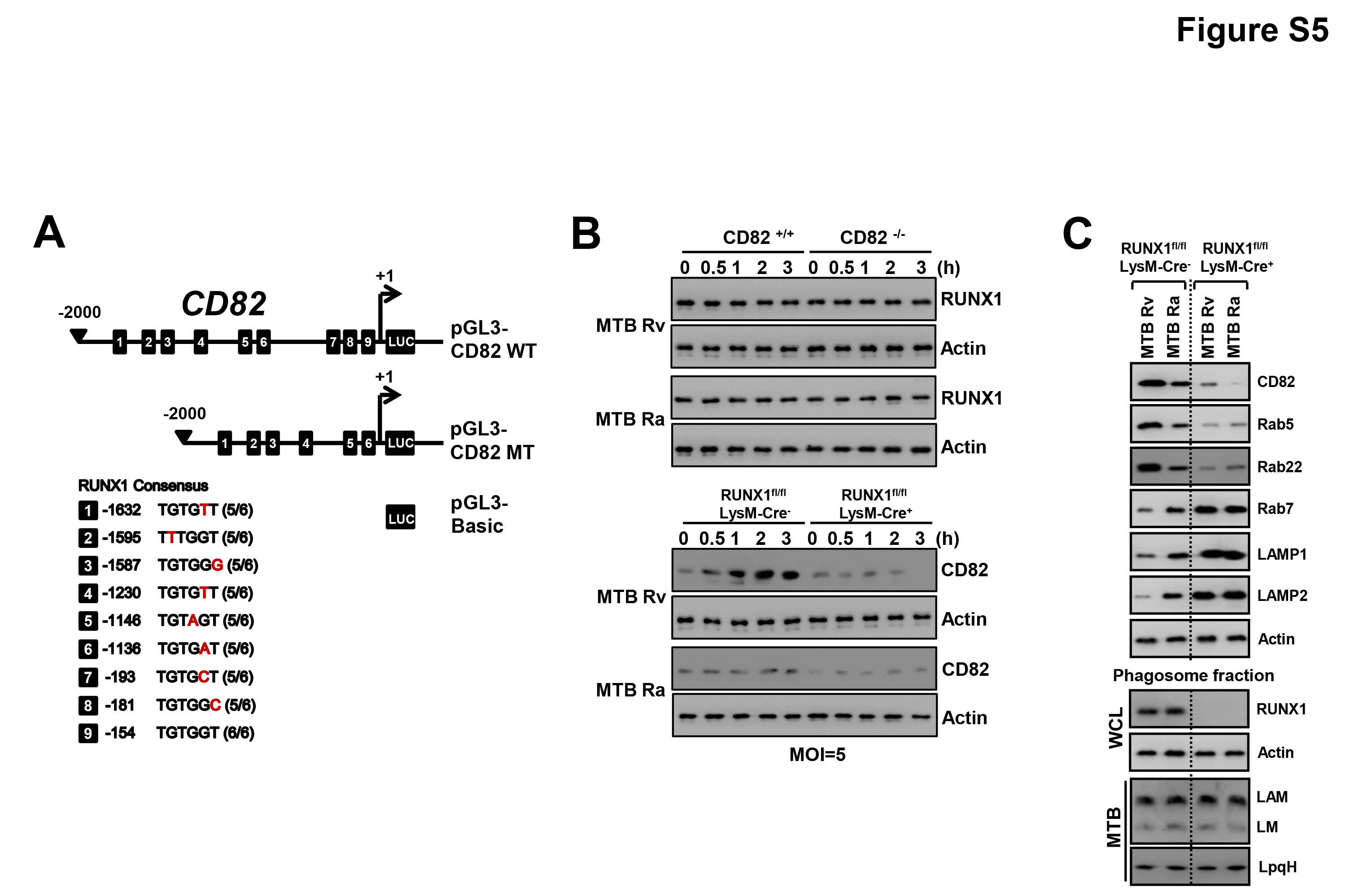
**

**Figure S6. CD82 associates with RUNX1 for CD82 expression and phagosome arrest by MTB Rv.** (**A**) Schematic representation of the mouse CD82 promoter-reporter constructs generation. Potential RUNX1 binding sites located in the 5′-flanking region of genes CD82 are shown as numbered boxes. Numbers in parentheses indicate the number of residues matching the consensus. n = A, T, G, or C. (**B**) BMDMs from CD82+/+ or CD82-/- (upper) or BMDMs from RUNX1^fl/fl^ LysM-Cre- or RUNX1^fl/fl^ LysM-Cre+ (lower) were infected with MTB Rv or MTB Ra (MOI=5) for the indicated times, followed by IB with αRUNX1 and αActin (upper) or with αCD82 and αActin (lower). (**C**) BMDMs from RUNX1^fl/fl^ LysM-Cre- or RUNX1^fl/fl^ LysM-Cre+ were infected with MTB Rv or MTB Ra (MOI=5) for 6 h. Mycobacteria-containing phagosome fractions were subsequently purified by sucrose-step-gradient-ultra-centrifugations, followed by IB to detect αCD82, αRab5, αRab22, αRab7, αLAMP1, αLAMP2, and αActin (upper) or IB with αRUNX1 and αActin (lower). The polyclonal MTB Ab and LpqH Ab detect the lipoglycans (LAM and LM) and lipoproteins (LpqH), respectively. The data are representative of three independent experiments with similar results (**B** and **C**).


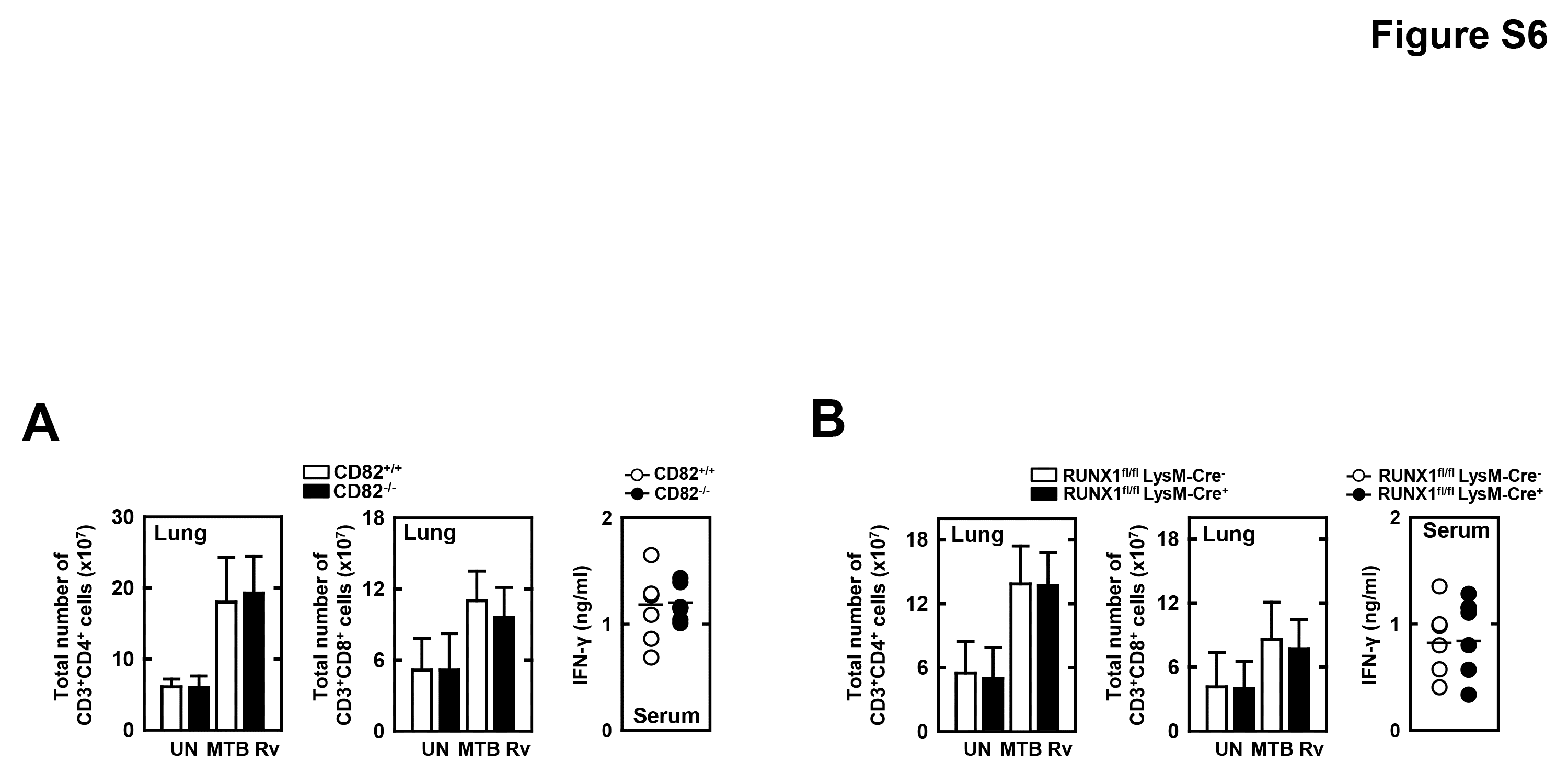


**Figure S7. CD82 and RUNX1-mediated T cell response.**

(**A** and **B**) Total cell number of CD3^+^CD4^+^ and CD3^+^CD8^+^ cells in lung and serum cytokine levels of IFNγ from MTB Rv-infected mice. Biological replicates (*n*=3) for each condition were performed.

**
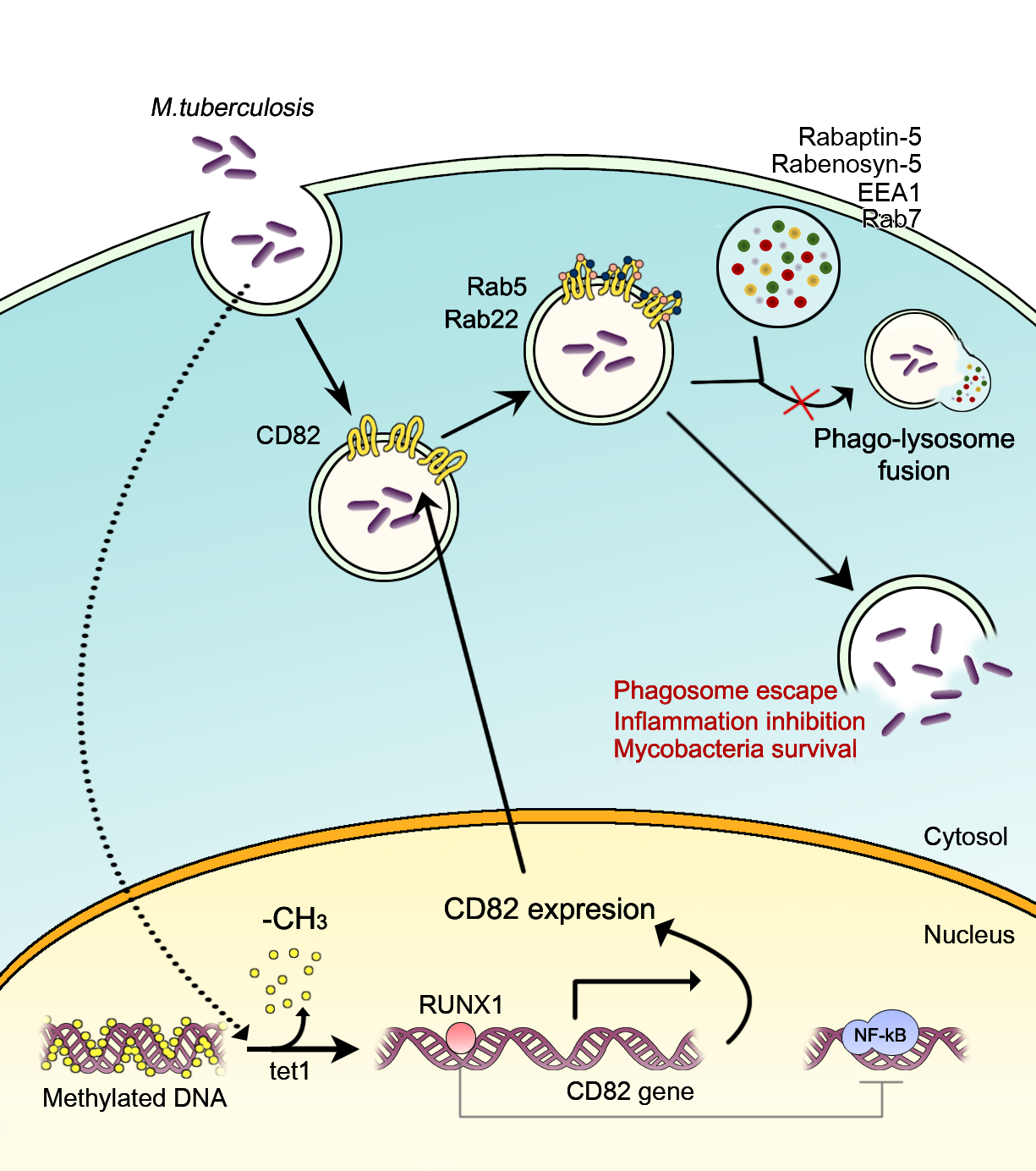
**

**Figure S8. Schematic models for the role of CD82 and CD82-RUNX1-Rab5/22-mediated mycobacteria virulence mechanisms during TB pathogenesis.**

Please see the discussion for detail.

**Supplemental Experimental Procedures**

*Antibodies*

Antibodies specific for actin (I-19), CD82 (C-16), RUNX1 (C-19), Rab5 (D-11), Rab22 (G-7), Rab7 (H-50), LAMP1 (E-5), LAMP2 (H4B4), Rabaptin-5 (B-8), YFP (FL), GFP (B-2), HA (F-7), Flag (D-8), and GST (B-14) were purchased from Santa Cruz Biotechnology. Specific antibodies against CD82 (ab66400), CD68 (ab955), Rabenosyn-5 ([ab190008)](http://www.abcam.com/rabenosyn-5-antibody-ab190008.html), EEA1 (ab2900), Histone H3 dimetyl K4 (ab7766), Histone H3 acetyl K14 (ab61232), Histone phospho S10 (ab5176), and Histone H4 acetyl K16 (ab109463) were from Abcam. Anti-*M. tuberculosis* polyclonal Ab (Gen-Way Biotech, San Diego, CA) and anti-LpqH clone IT-19 (hybridoma from BEI Resources, Manassas, VA) were used.

*Protein purification and mass spectrometry*

Precipitates were washed extensively with lysis buffer. Proteins bound to beads were eluted and separated on a NuPAGE 4-12% Bis-Tris gradient gel (Life Technologies). After silver staining (Life Technologies), specific protein bands were excised and analysed by LC-MS/MS at the Korea Research Institute of Bioscience and Biotechnology Mass Spectrometry facility, and amino acid sequences were determined by tandem mass spectrometry and database searches

*Immunoblot analysis and Immunoprecipitation*

For Western blot analysis, primary Abs were used at a 1/1,000 dilution. For the immunoprecipitation, cells were harvested and lysed in NP-40 buffer supplemented with a complete protease inhibitor cocktail (Roche). The lysates were mixed and precipitated with Abs and protein A-Sepharose by incubation at 4°C for 18 h on a rotator. For GST Pulldown assays, pre-cleared lysates were mixed with 50% slurry of glutathione-conjugated Sepharose beads (Amersham Biosciences), and the binding reactionwas incubated for 4 h at 4°C. The samples were subsequently solubilized in SDS sample buffer and separated by SDS-PAGE for Western blot analysis. Antibody binding was visualized by chemiluminescence (ECL;Millipore) and detected by a Vilber chemiluminescence analyzer (Fusion SL 3;Vilber Lourmat).

*Isolation of MTB-containing phagosomes*

Eight 15-cm^2^ plates of BMDMs were used for each condition, bacteria at an MOI of 10 were infected to macrophages for 2-4 h and washed. Infected cells were collected, lysed, stepwise sucrose gradient centrifugation, and subjected to fractionation as described previously [^2^](#_ENREF_2). The phagosomal fractions were extracted by the cell lysis buffer containing 25 mM Tris–HCl pH 7.6, 150 mM NaCl, 1% Nonidet P-40, 1% sodium deoxycholate and 0.1% SDS. We confirmed that mycobacterial proteins are not extracted by the cell lysis buffer as described previously [^3^](#_ENREF_3). For immunoblotting analysis, aliquots of 20 μg of phagosomal fractions were separated by SDS–PAGE and then subjected to immunoblotting analysis

*CFU assay*

To assay bacterial viability within macrophages, cells were infected with MTB for 4 h and then washed with PBS to remove extracellular bacteria. Thereafter, the infected cells were incubated for the indicated time periods. Finally, cells were harvested and lysed with 0.3% saponin (Sigma-Aldrich) to release the intracellular bacteria, and cell lysates were then resuspended vigorously, transferred to screw cap tubes, and sonicated in a preheated 37°C water bath sonicator (Elma) for 5 min. Aliquots of the sonicates were then diluted 10-fold in Middlebrook 7H9 medium. Four dilutions of each sample were plated separately on Middlebrook 7H10 agar plates and incubated at 37°C with for 2–3 weeks

*Quantitative real-time PCR*

Total RNA was extracted from cells using the RNeasy RNA extraction Mini-Kit (Qiagen). cDNA was synthesized using the Enzynomix kit (Enzynomix) and quantitative PCR was performed using gene-specific primer sets (Bioneer) and SYBR Green PCR Master Mix (Roche). Real-time PCR was performed using a QuantStudio™ 3 (ABI), according to the manufacturer’s instructions. Data were normalized against β-actin expression. Relative expression was calculated using the delta-delta CT method. The sequences of the primers used are listed in **Supplementary Table 1**.

*Measurements of cytokines*

Mouse cytokines in culture supernatants were measured by a BD OptEIA ELISA set (BD Pharmingen) as described previously [^4^](#_ENREF_4). All assays were performed as recommended by the manufacturers.

*Lentiviral shRNA production and transduction*

Lentiviral shRNA production, concentration, titration and transduction were described previously using the target shRNA plasmid DNA (mouse CD82, RMM4532-EG12521; mouse Rab5, RMM4532-EG271457; Rab22, RHS5086-EG19334) were purchased from Open Biosystems. A parallel experiment using a GFP-encoding lentivirus (the pGIPZ lentiviral vector; Open Biosystems) indicated that 80% of cells were successfully transduced by the virus.

*Luciferase assays*

J774A.1 cells were transiently transfected with a luciferase gene under the control of the pGL3 promoter-containing CD82 sites, or the RUNX1 transcription recognition sequences were used. We also used the pRL vector expressing a Renilla luciferase gene (Promega, E2241) for constitutive protein expression, and the dual luciferase reporter assay system (Promega, E1910) substrate. For luciferase reporter gene assays, mouse CD82 promoter constructs were used. For the mouse CD82 promoter construct, a 2000-bp fragment upstream of the predicted transcription start site of the mouse CD82 gene was generated by PCR amplification from mouse genomic DNA using the sense primer 5′-TGGTCTAACCTGGTCTAATCTGG-3′ and antisense primer 5′-AAGACCACGCCCTTGACAC-3′. These fragments were digested with *Mlu I* and *Bgl II* and cloned upstream of the luciferase gene in the pGL3-Basic reporter vector (Promega). Searches for putative DNA binding sites for transcription factors in the mouse CD82 5′-flanking region sequences were conducted using TFSEARCH (http://www.cbrc.jp/research/db/TFSEARCH.html) and MOTIF Search ([http://motif.genome.jp](http://motif.genome.jp/)). A schematic representation of the generation of mouse CD82 promoter reporter constructs is shown in **Supplementary Figure 5A**. The reporter gene assay was performed as described previously [^6^](#_ENREF_6). J774A.1 cells were plated in 6-well culture plates and transfected with a firefly reporter vector (4.0 μg) and pRL vector expressing the Renilla luciferase gene (0.1 μg), together with the indicated expression plasmids (4.0 μg) using the Lipofectamine 3000 (Invitrogen) reagent according to the manufacturer’s instructions. After 2 day of transfection, cells were lysed in Passive Reporter Lysis Buffer (Promega, E1910), and luciferase activity was measured. Firefly luciferase activity was normalized to Renilla luciferase activity. Each transfection was performed in triplicate, and 3 independent experiments were conducted.

*Methylation-Specific Polymerase Chain Reaction (MS-PCR)*

Genomic DNA was isolated from cells using a DOKDO Prep™ Blood Genomic DNA Purification Kit (Elpis Biotech, Daejeon, Korea) according to the manufacturer’s instructions. Bisulfite modification of genomic DNA samples was processed using the EZ DNA Methylation™ Kit (Zymo Research). Methylation-specific primers were designed using MethPrimer (http://www.urogene.org/cgi-bin/methprimer/methprimer.cgi) in **Supplementary Table 1**. The primers used to differentiate methylated and unmethylated CpG sites in the promoter of the CD82 gene. MS-PCR was performed using an EpiScope® MSP Kit (TaKaRa Bio Inc., Kyoto, Japan). PCR products were electrophoresed on a 1% agarose gel pre-mixed with NEOgreen (NeoScience Co., Ltd., Suwon) and then imaged by a NaBI nucleic acid gel imaging system (NeoScience Co., Ltd., Suwon, Korea).

*Sodium Bisulfite modification for pyrosequencing analysis*

Bisulfite modified gDNA was prepared using EZ DNA Methylation-Lightning^TM^ kit (Zymo Research, USA) according to the manufacturer’s instructions. The bisulfite reaction was carried out on 500 ng gDNA and the reaction volume was adjusted to 20ul with sterile water and 130ul of CT conversion Reagent were added. The sample tubes were placed in a thermal cycler (MJ Research) and performed the following steps : 8min at 98℃, 60min at 54°C, and stored at 4°C for up to 20 hrs.

The DNA was purified using reagent contained in EZ DNA Methylation- Lightning^TM^ kit (Zymo Research, USA). The converted samples were added into Zymo-Spin IC^TM^ Column containing 600 ul of the M-Binding Buffer and mixed by inverting the column several times. The column was centrifuged at full speed for 30sec and discarded the folw-through. The column was washed by adding 200ul of M-Wash Buffer and spined at full speed and then 200 ul of M-Desulphonation Buffer was added to the column and let stand at room temperature (20-30°C) for 15-20min. After incubation, the column was centrifuged at full speed for 30sec. The column was washed by adding 200ul of M-Wash Buffer and spined at full speed (repeat this step). The converted gDNA was eluted by adding 20ul of M-Elution Buffer into the column and spin. DNA samples were finally stored at -20°C until further use.

*Pyrosequencing analysis*

We used the bisulfite pyrosequenicng method for methylation analyses of the CD82 gene. Each primer was designed using Pyrosequencing Assay Design Software v2.0 (Qiagen). PCR reaction was carried out in a volume of 20ul with 20ng or more converted gDNA, PCR premixture (Enzynomics, Korea), 1ul of 10pmole/ul Primer-S, and 1ul of 10pmole/ul biotinylated-Primer-As. The amplification was carried out according to the general guidelines suggested by Pyrosequencing: denaturating at 95℃ for 10min, followed by 45 cycles at 95°C for 30sec, at 58°C for 30sec, at 72°C for 30sec and a final extension at 72°C for 5min. The PCR reaction (2ul) was confirmed by electrophoresis in a 2% Agarose gel and visualized by ethidium bromide staining.

ssDNA template was prepared from 16-18ul biotinylated PCR product using streptavidin Sepharose® HP beads (Amersham Biosciences, Sweden) following the PSQ 96 sample preparation guide using multichannel pipets. Fifteen picomoles of the respective sequencing primer sere added for analysis. Sequencing was performed on a PyroMark ID system with the Pyro Gold reagents kit (Qiagen) according to the manufacturer’s instruction without further optimization. The methylation percentage was calculated by the average of the degree of methylation at -677~-80 CpG sites formulated in pyrosequencing.

| **Targets CpG islands and the primers for pyrosequencing** | | | |
| --- | --- | --- | --- |
| Gene | Primer | | Size(bp) |
| *CD82* | Forward | 5’- GGAGGGTTGGTAAAGGGTAGTTA-3’ | 137 |
|  | Biotinylated-reverse | 5’biotin- AACACCCCCCCTATACAATTCCCCTCT-3’ |  |
|  | Sequencing primer | 5’- AGTTTAGTGTTGTGTTAAGG-3’ |  |

*Chromatin immunoprecipitation (ChIP) assay*

ChIP experiments were performed using a protocol adapted from Upstate. Chromatin from 1 × 10^7^ cells was used for each immunoprecipitation. BMDMs were collected and resuspended in digestion buffer (50 mM Tris-Cl, pH 7.6; 1 mM CaCl2, 0.2% Triton X-100, 5 mM sodium butyrate, 1X protease inhibitor cocktail, 0.5 mM PMSF). Sonicate the lysate to shear DNA fragments ranging in size from 100–500 bp and keep the samples on ice. We used Bioruptor sonicator for four cycles of 10 minutes, with 30 seconds on/off. After sonication, the cells were incubated with RIPA buffer (10 mM Tris, pH 7.4, 1 mM EDTA, 0.1% SDS, 0.1% Sodium deoxycholate, 1% Triton X-100) and subjected to immunoprecipitation with antibodies against RUNX1 (Santa Cruz, C-19) and normal mouse IgG (Santa Cruz Biotechnology, sc-2025) used as a control with Dynabeads Protein A beads (Invitrogen) for 16 hr at 4 °C. The DNA was extracted and purified following Upstate’s instruction and used in regular PCR analysis. The chromatin immunoprecipitates for the proteins and RUNX1 marks were analyzed using regular PCR, with one modification; the cDNA was replaced with immunoprecipitated DNA and normalized by input DNA. Primers used for ChIP-PCR are listed in **Supplementary Table 1**.

*Immunohistostaining*

For immunohistostaining of tissue sections, lungs were fixed in 10% formalin and sectioned in paraffin, as previously described [^7^](#_ENREF_7). To examine CD82 or RUNX1 expression, 4-μm paraffin sections were deparaffinized and hydrated by serially dipping into 100–70% ethanol, distilled water, and PBS. The slides were antigen retrieved in sodium citrate buffer and blocked for 20 min in 1.5% normal rabbit serum in PBS and stained for CD82 or RUNX1 (Santa Cruz) for 18 h at 4 °C. After washing, the appropriate fluorescently labeled secondary Abs was incubated for 1 h at 25 °C. Slides were examined using laser-scanning confocal microscopy (model LSM 800; Zeiss). For colocalization analysis, the co-distribution of the CD82 and macrophages marker CD68 were quantified and validated statistically by Pearson coefficient, as specified by the ZEN 2009 software (version 5.5 SP1; Zeiss).

Staining intensity of CD82 or RUNX1 was scored independently by pathologists in a blinded manner with 0 (no staining), 1 (weak staining), 2 (intermediate staining), or 3 (strong staining) for each sample (right). Data shown are the mean ± SD of five experiments.

CD82 or RUNX1-stained area in CD68-stained total cell area (%) was quantified according to the following procedure: digital exclusion of tissue artifacts; threshold for the intensity of positive staining (DAB); threshold for the total cell area by including both positive (DAB) and control (AEC) staining; and application of a determined setting for 10-15 fields/tissue section. Data are obtained as the mean percentage of positively stained area (CD82 or RUNX1) within the total cell area of a specific marker (CD68) and given as medians with interquartile range (n=5).

*Flow cytometry analysis of lung cells*

(**A**) CD82^+/+^ and CD82^-/-^ mice (*n*=35) or (**B**) RUNX1^fl/fl^ LysM-Cre+ and RUNX1^fl/fl^ LysM-Cre- mice were *i.v.* injected with MTB (1×10^8^ CFU/mouse). After 3 wks of infection, mice were sacrificed and the lungs were harvested to obtain single-cell suspensions. The samples were then filtered through a 70-μm filter and used for flow cytometry analysis. Single-cell suspensions were pretreated with fluorescently labeled antibodies. Purified anti-mouse-CD8 (clone 53-6.7), anti-mouse-CD4 (clone GK1.5) and anti-mouse-CD3 (clone OKT3) were obtained from eBioscience. After two washes with PBS, cells were fixed in 4% paraformaldehyde and immediately analysed. In each case, 10,000 cells were acquired. The data were analyzed using BD FACSCanto II (Becton Dickinson) using Flowjo software (Tree Star, Inc.).

*Lung Histopathology*

Serial sections (4 μm) were stained with hematoxylin and eosin (H&E). A semiquantitative histopathologic scoring system was developed on the basis of the presence and abundance of the following: (*1*) perivascular edema (0, absent; 1, mild to moderate, involving fewer than 25% of the perivascular spaces; 2, moderate to severe, involving more than 25% but less than 75% of perivascular spaces; or 3, severe, involving more than 75% of perivascular spaces); (*2*) perivascular/peribronchial acute inflammation (0, absent; 1, mild acute inflammation in the perivascular edematous space, with fewer than 5 neutrophils per high-power field [hpf]; 2, moderate acute inflammation in the perivascular spaces, extending to involve the peribronchial spaces, with more than 5 neutrophils per hpf in these regions; or 3, severe, acute inflammation in the perivascular and peribronchial spaces with numerous neutrophils encircling most [50%] of bronchioles); (*3*) goblet-cell metaplasia of bronchioles (0, absent; 1, few goblet cells present in one or two bronchiolar profiles; or 2, large numbers of goblet cells present); and (*4*) eosinophilic macrophages in alveolar spaces (0, absent; 1, present in fewer than 25% of alveolar spaces; or 2, present in 25% of alveolar spaces). A total inflammatory scores (range 0 to 10), taken as the sum of the individual scores, was determined by a pulmonary pathologist who was blinded to genotype and treatment-group assignment.

**Supplemental References**

1. Singer BD, Mock JR, D'Alessio FR, Aggarwal NR, Mandke P, Johnston L *et al.* Flow-cytometric method for simultaneous analysis of mouse lung epithelial, endothelial, and hematopoietic lineage cells. *Am J Physiol Lung Cell Mol Physiol* 2016; **310:** L796-801.

2. Beatty WL, Rhoades ER, Hsu DK, Liu FT, Russell DG. Association of a macrophage galactoside-binding protein with Mycobacterium-containing phagosomes. *Cell Microbiol* 2002; **4:** 167-176.

3. Lee BY, Jethwaney D, Schilling B, Clemens DL, Gibson BW, Horwitz MA. The Mycobacterium bovis bacille Calmette-Guerin phagosome proteome. *Mol Cell Proteomics* 2010; **9:** 32-53.

4. Yang CS, Lee JS, Rodgers M, Min CK, Lee JY, Kim HJ *et al.* Autophagy protein Rubicon mediates phagocytic NADPH oxidase activation in response to microbial infection or TLR stimulation. *Cell Host Microbe* 2012; **11:** 264-276.

5. Yang CS, Yuk JM, Lee YH, Jo EK. Toxoplasma gondii GRA7-Induced TRAF6 Activation Contributes to Host Protective Immunity. *Infect Immun* 2015; **84:** 339-350.

6. Yang CS, Kim JJ, Lee HM, Jin HS, Lee SH, Park JH *et al.* The AMPK-PPARGC1A pathway is required for antimicrobial host defense through activation of autophagy. *Autophagy* 2014; **10:** 785-802.

7. Yang CS, Yuk JM, Kim JJ, Hwang JH, Lee CH, Kim JM *et al.* Small heterodimer partner-targeting therapy inhibits systemic inflammatory responses through mitochondrial uncoupling protein 2. *PLoS One* 2013; **8:** e63435.
